# Different DNA methylome, transcriptome and histological features in uterine fibroids with and without MED12 mutations

**DOI:** 10.1038/s41598-022-12899-7

**Published:** 2022-05-26

**Authors:** Ryo Maekawa, Shun Sato, Tetsuro Tamehisa, Takahiro Sakai, Takuya Kajimura, Kotaro Sueoka, Norihiro Sugino

**Affiliations:** Department of Obstetrics and Gynecology, Yamaguchi University Graduate School of Medicine, Ube, 755-8505 Japan

**Keywords:** Genetics research, Molecular medicine, Pathogenesis, Reproductive disorders

## Abstract

Somatic mutations in Mediator complex subunit 12 (MED12m) have been reported as a biomarker of uterine fibroids (UFs). However, the role of MED12m is still unclear in the pathogenesis of UFs. Therefore, we investigated the differences in DNA methylome, transcriptome, and histological features between MED12m-positive and -negative UFs. DNA methylomes and transcriptomes were obtained from MED12m-positive and -negative UFs and myometrium, and hierarchically clustered. Differentially expressed genes in comparison with the myometrium and co-expressed genes detected by weighted gene co-expression network analysis were subjected to gene ontology enrichment analyses. The amounts of collagen fibers and the number of blood vessels and smooth muscle cells were histologically evaluated. Hierarchical clustering based on DNA methylation clearly separated the myometrium, MED12m-positive, and MED12m-negative UFs. MED12m-positive UFs had the increased activities of extracellular matrix formation, whereas MED12m-negative UFs had the increased angiogenic activities and smooth muscle cell proliferation. The MED12m-positive and -negative UFs had different DNA methylation, gene expression, and histological features. The MED12m-positive UFs form the tumor with a rich extracellular matrix and poor blood vessels and smooth muscle cells compared to the MED12m-negative UFs, suggesting MED12 mutations affect the tissue composition of UFs.

## Introduction

Uterine fibroids are tumors derived from uterine smooth muscle cells and are most common in gynecologic neoplasms^1^. In the last decade, somatic mutations of Mediator complex subunit 12 (MED12) have been found to be reliable biomarkers of uterine fibroids^2–4^. MED12 is located on the X chromosome and encodes the RNA polymerase II mediator complex and part of the transcriptional preinitiation machinery. Mutations of MED12, especially mutations in exon 2, are thought to be the underlying causes of about 70% of human uterine fibroids^2,5,6^. However, the remaining 30% of uterine fibroids do not have MED12 mutations, which indicates that the role of MED12 mutations in the pathogenesis of uterine fibroids is unclear.

Uterine fibroids differ in size and the number of nodules. Uterine fibroids carrying MED12 mutations are reported to be smaller and often more numerous than those without MED12 mutations^4^. Several reports have suggested links between MED12 mutations and different phenotypes of uterine fibroids. Uterine fibroids without MED12 mutations were found to have elevated erythropoietin expression in an estrogen-dependent manner, while the uterine fibroids with MED12 mutation had low erythropoietin^7,8^. Furthermore, uterine fibroids with and without MED12 mutations had different cell components and different amounts of collagen^9^. These reports suggest that MED12 mutations are associated with different phenotypes of uterine fibroids. On the other hand, genome-wide gene expression profiles were not different between uterine fibroids with and without MED12 mutations^10^. A comparison of transcriptomes from uterine fibroids with and without MED12 mutations found difference in only a few specific intracellular signaling-pathways including arachidonic acid metabolism^11^. Thus, it remains unclear whether uterine fibroid phenotypes are associated with MED12 mutations.

DNA methylation is a major type of epigenetic mark. DNA methylation profiles define each type of normal cells and distinguish cell types^12–14^, and therefore have been used to characterize abnormal cells^13,14^. DNA methylation is tissue/cell-specific, and DNA methylation profiling is more useful than profiling mRNA expression to define the cell identity^15^. We previously reported that uterine fibroids had different DNA methylation profiles from normal myometrium by genome-wide approach and that DNA methylation profiles segregated the uterine fibroids and normal myometrium^15,16^. Furthermore, using these differently methylated genes between uterine fibroids and normal myometrium, we found some potential mechanisms for the pathogenesis of uterine fibroids^15–19^. We also found significant differences in DNA methylation levels of those genes between uterine fibroids with and without MED12 mutations^16^. These findings led us to investigate differences in genome-wide DNA methylation profiles between uterine fibroids with and without MED12 mutations.

We recently identified SATB2 and NRG1 as potential upstream regulatory factors in uterine fibroids^19^. SATB2 and NRG1 expressions were increased in uterine fibroids compared to the myometrium. Both SATB2 and NRG1 activated WNT/beta-catenin and TGF-beta signaling pathways, which are related to the pathogenesis of uterine leiomyomas^19^. Interestingly, established cell lines overexpressing SATB2 morphologically changed from spindle-like forms to fibroblast-like forms with elongated protrusions^19^, suggesting that SATB2 and NRG2 play essential roles in initiating tumorigenesis in uterine fibroids. However, the association between the expression of NRG1 and SATB2, and MED12 mutations is still unclear.

In the present study, we examined the associations between MED12 mutations and four aspects of uterine fibroids: (1) genome wide methylation profiles, (2) cellular functions as revealed by transcriptome analyses, (3) different histopathological features and (4) the expressions of NRG1 and SATB2.

## Results

### Hierarchical clustering using DNA methylation profiles

**Figure 1:**
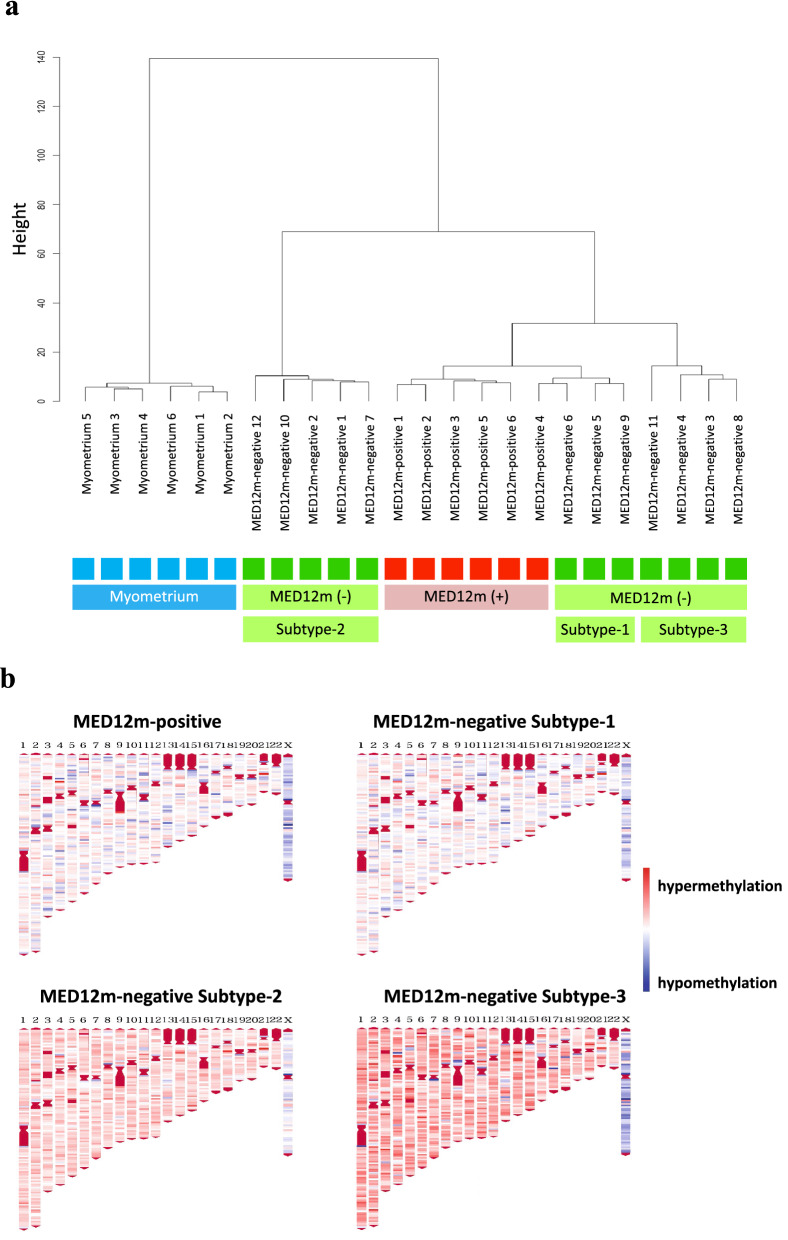
DNA methylation profiling of the MED12m-positive and -negative uterine fibroids, and myometrium. (**a**) DNA methylation profiles of the MED12m-positive and -negative uterine fibroids, and myometrium were compared using hierarchical clustering analyses. Distances of DNA methylation pattern are indicated as height. Each color indicates the myometrium (light blue), the MED12m-positive uterine fibroids (red), and the MED12m-negative uterine fibroids (green). The MED12m-negative uterine fibroids were further classified into three different clusters, Subtype-1, Subtye-2, and Subtype-3. (**b**) Chromosomal distribution of hyper- or hypomethylated CpGs in the MED12m-positive and -negative uterine fibroids (Subtype-1, -2, and -3) compared to the myometrium are shown. The locations of CpG sites, which have p < 0.05 and beta-value difference > 0.2 compared to the myometrium, are indicated with red (hypermethylated CpGs) or blue (hypomethylated CpGs). Autosomal and sex chromosome numbers are shown on the top.

We first examined the DNA methylome of the uterine fibroids with and without MED12 mutations (MED12m-positive uterine fibroids (n = 6) and MED12m-negative uterine fibroids (n = 12), respectively), and myometrium (n = 6). Hierarchical clustering showed that the myometrium made a distinct cluster from uterine fibroids (Fig. 1a). The MED12m-positive and -negative uterine fibroids were classified into different clusters (Fig. 1a), suggesting that uterine fibroids with and without MED12 mutations are different at the molecular levels.

The MED12m-positive uterine fibroids were clearly clustered, while the MED12m-negative uterine fibroids can be further classified into three clusters (Subtype-1, -2, and -3; Fig. 1a). Subtype-1 was classified into the same cluster as the MED12m-positive uterine fibroids (Fig. 1a). Subtypes-2 and -3 were classified into clusters different from Subtype-1 (Fig. 1a).

Figure 1b shows the distribution of aberrantly methylated CpGs in the MED12m-positive and -negative uterine fibroids compared to the myometrium throughout the chromosomes. The results showed that the DNA methylation statuses of Subtype-1 are similar to that of the MED12m-positive uterine fibroids. On the other hand, in Subtypes-2 and -3, the DNA methylation status in the autosomes tended to be hypermethylated compared to that in Subtype-1 and the MED12-positive uterine fibroids (Fig. 1a).

### Differentially expressed genes (DEGs)

We determined DEGs in the MED12m-positive and -negative uterine fibroids compared to the myometrium. The MED12m-positive fibroids had 157 increased and 233 decreased genes compared to the myometrium (Supplementary Tables S1 and S2 online). The MED12m-negative fibroid had 110 increased and 207 decreased genes compared to the myometrium (Supplementary Tables S3 and S4 online). The DEGs were subjected to the GO enrichment analysis to know the characteristics of the DEGs.

**Figure 2:**
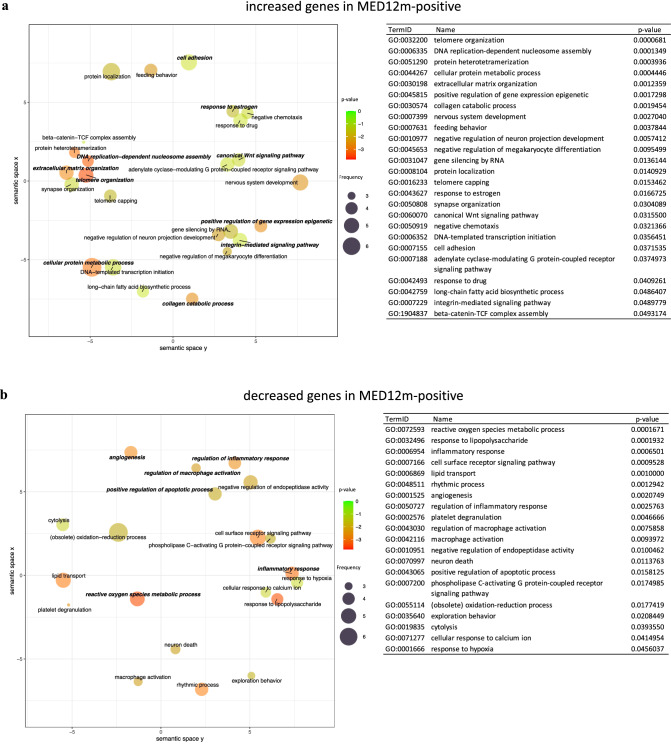

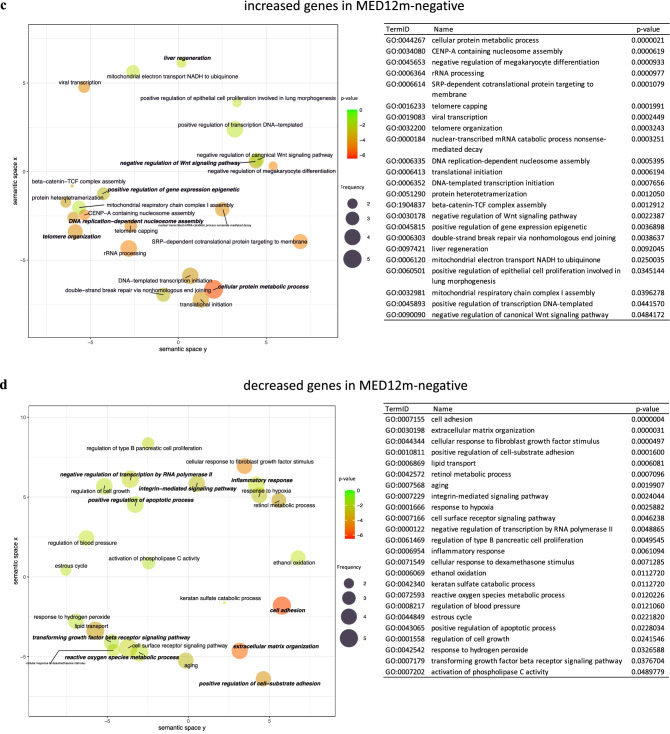

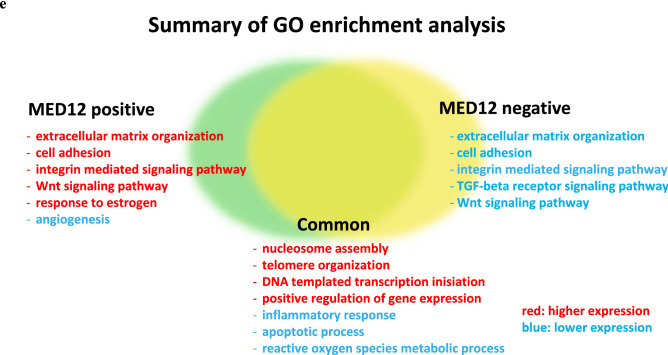
The scatterplot of GO terms in DEGs. The plots and tables show the GO terms after the redundancy reduction in the MED12m-positive-increased (**a**), -decreased (**b**), the MED12m-negative-increased (**c**), and -decreased (**d**) DEGs. The colors indicate the log10(p-value) of the summarized GO terms. The size of the circle indicates the frequency of the GO term in the underlying GO database. The circles of more general terms are plotted larger. The color and the size of circles are plotted according to the default setting of REVIGO^39^. (**e**) Summary of GO analysis in the MED12m-positive and -negative uterine fibroids (drawn with Microsoft PowerPoint). The specific terms to the MED12m-positive uterine fibroids, the MED12m-negative uterine fibroids, and commonly detected terms are shown. Red and blue mean higher and lower expression compared to the myometrium.

The GO terms "telomere organization", "DNA replication-dependent nucleosome assembly", "positive regulation of gene expression epigenetic", "extracellular matrix organization", "collagen catabolic process", "cell adhesion", "integrin-mediated signaling pathway", "cellular protein metabolic process", "response to estrogen", and "canonical Wnt signaling pathway" were detected in the increased genes in the MED12m-positive uterine fibroids (Fig. 2a). In the decreased genes in the MED12m-positive uterine fibroids (Fig. 2b), the GO terms "reactive oxygen species metabolic process", "inflammatory response", "regulation of inflammatory response", "angiogenesis", "regulation of macrophage activation", and "positive regulation of apoptotic process" were detected.

In the MED12m-negative uterine fibroids, the GO terms "cellular protein metabolic process", "telomere organization", "DNA replication-dependent nucleosome assembly", "positive regulation of gene expression epigenetic", "liver regeneration", and "negative regulation of canonical Wnt signaling pathway" were detected in the increased genes (Fig. 2d). In the decreased genes in the MED12m-negative uterine fibroids (Fig. 2d), the GO terms "cell adhesion", "extracellular matrix organization", "positive regulation of cell-substrate adhesion", "integrin-mediated signaling pathway", "negative regulation of transcription by RNA polymerase II", "inflammatory response", "reactive oxygen species metabolic process", "positive regulation of apoptotic process", and "transforming growth factor-beta receptor signaling pathway" were detected.

Figure 2e summarizes the results of GO enrichment analyses of DEGs. Compared to myometrium, MED12m-positive uterine fibroids showed increased activities of extracellular matrix organization, cell adhesion, integrin-mediated signaling, and Wnt signaling pathway, whereas MED12m-negative uterine fibroids had the decreased activities of them and TGF-beta signaling, suggesting that MED12m-positive uterine fibroids have the increased activity of extracellular matrix formation compared with MED12m-negative uterine fibroids. In addition, MED12m-positive uterine fibroids showed the increased responsiveness to estrogen and decreased angiogenic activities. Both types of uterine fibroids had increased cell proliferation and transcription activities and decreased inflammatory response and reactive oxygen species metabolic process activities compared to myometrium.

**Figure 3:**
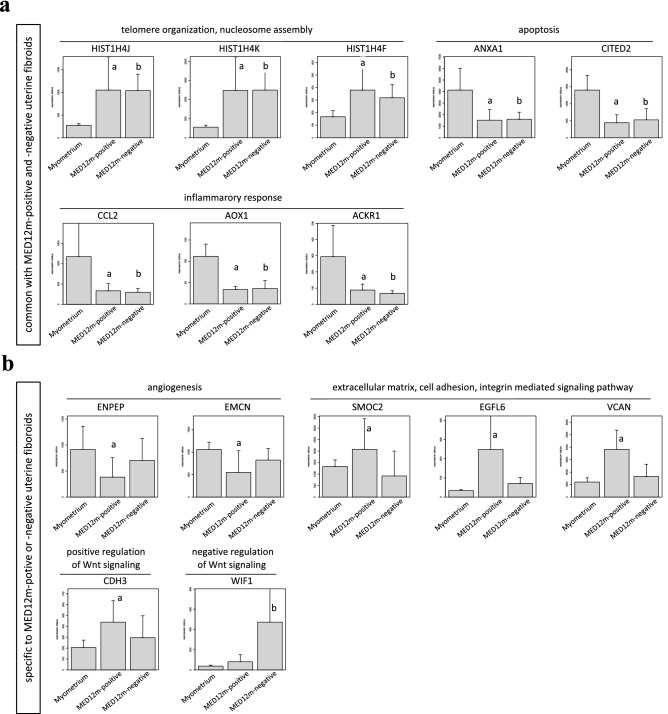
Expression levels of representative genes in the detected biophysical processes. According to Figs. 2e, Fig. 3 shows the expression statuses of the representative genes not only from the commonly detected terms between the MED12m-positive and MED12m-negative uterine fibroids (**a**), but also from the terms specific to the MED12m-positive or the MED12m-negative uterine fibroids (**b**). We selected the genes in which the difference in expression levels was clearly significant among the MED12m-positive uterine fibroids, the MED12m-negative uterine fibroids, and the normal myometrium. ^a^p < 0.01 (myometrium (n = 6) vs. MED12m-positive uterine fibroids (n = 6), student t-test). ^b^p < 0.01 (myometrium (n = 6) vs. MED12m-negative uterine fibroids (n = 9), student t-test).

According to Figs. 2e, 3 shows the expression statuses of the representative genes not only from the commonly detected terms between the MED12m-positive and MED12m-negative uterine fibroids (Fig. 3a), but also from the terms specific to the MED12m-positive or the MED12m-negative uterine fibroids (Fig. 3b). We selected the genes in which the difference in expression levels was clearly significant among the MED12m-positive uterine fibroids, the MED12m-negative uterine fibroids, and the normal myometrium.

### Genes whose promoters are differentially methylated

We focused on the genes whose promoters are differentially methylated because DNA methylation on the promoter regions is closely associated with gene expression. We first investigated the genes whose promoters are differentially methylated between the MED12m-positive and MED12m-negative uterine fibroids. There were 25 hypermethylated and 73 hypomethylated genes in the MED12m-positive uterine fibroids compared to the MED12m-negative uterine fibroids (Supplementary Table S5 online). GO analysis with the 73 hypomethylated genes in the MED12m-positive uterine fibroids showed the GO term "cell differentiation" including SMAD3, MEF2D, ELF1, and MICAL2, which are associated with TGF-beta signaling, and COL6A3. On the other hand, no significant GO term was extracted for the 25 hypermethylated genes in the MED12m-positive uterine fibroids. These findings are consistent with the data on the gene expression (Figs. 2) indicating that TGF-beta signaling and extracellular matrix organization are more activated in the MED12m-positive uterine fibroids compared with the MED12m-negative uterine fibroids. It is suggested that different DNA methylation on the promoters between MED12m-positive and MED12m-negative uterine fibroids partially contributes to the different characters of MED12m-positive and MED12m-negative uterine fibroids.

We next investigated the genes whose promoters are differentially methylated among the three subtypes of the MED12m-negative uterine fibroids. As shown in Supplementary Table S6 online, there were a few genes whose promoters are differentially methylated among them, and no significant GO term was extracted. This result suggests that DNA methylation on the other regions than promoters is rather associated with subtyping in the MED12m-negative uterine fibroids.

### Weighted gene co-expression network analysis (WGCNA)

The preceding DEGs (Fig. 2) is based on comparing the uterine fibroids with the myometrium, and this analytic approach has been used so far^10,11^. In general, in the analytic method that compares the target tissues to the control tissues, there is a possibility of missing the essential character of the target tissue when the cell character of the target tissue is close to that of the control tissue. Therefore, to know the intrinsic character in each of the MED12m-positive and -negative uterine fibroids, we used a WGCNA analysis^20,21^. WGCNA is a system biology method to describe the correlation patterns among the genes across microarray samples such as transcriptome data and to find the groups with highly correlated genes that work in the same biological functions^20,21^. We defined groups consisting of highly correlated genes as co-expressed gene (COG) groups and detected unique properties in the MED12m-positive and -negative uterine fibroids by comparing the intrinsic functions of each tissue.

The transcriptome data of the MED12m-positive and -negative uterine fibroids were independently subjected to the WGCNA. In the MED12m-positive and -negative uterine fibroids, WGCNA identified 26 and 14 COG groups, respectively (Table 1), and these genes were subjected to the GO enrichment analysis.

**Table 1:** COGs groups detected by WGCNA and the numbers of COGs groups with significant KEGG pathways and GO terms.

	Total genes	Genes assigned to COGs groups	Genes without assignment to COGs groups	Number of detected COGs groups	Number COGs groups with significant GO terms
MED12m-positive	19,860	19,859	1	26	3
MED12m-negative	19,860	16,183	3677	14	5

**Figure 4:**
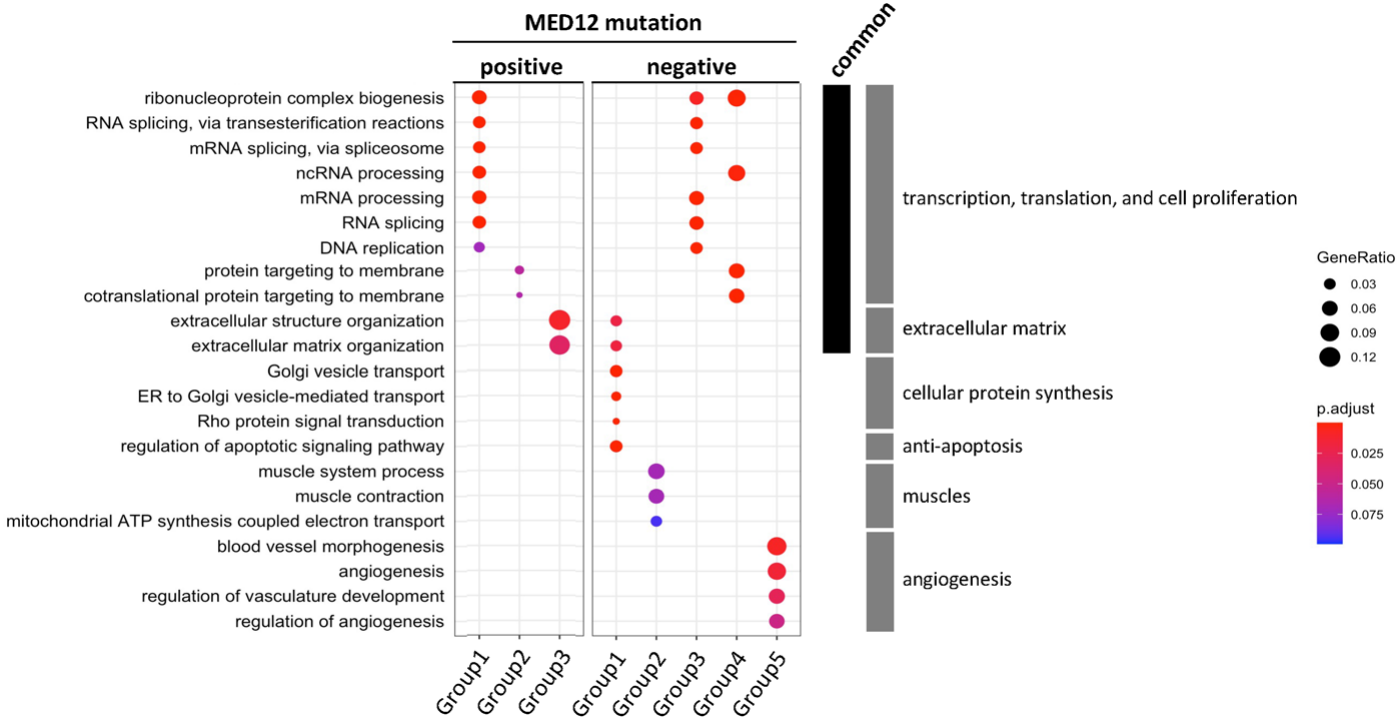
Enriched GO terms identified by weighted gene co-expression network analysis (WGCNA) in the MED12m-positive and -negative uterine fibroids. Twenty-six and 14 COGs groups in the MED12m-positive and -negative uterine fibroids, respectively, were introduced into the pathway and GO enrichment analyses. Three and five COGs groups in the MED12m-positive and -negative uterine fibroids were significantly enriched with GO terms. The other 23 and 9 COGs groups in the MED12m-positive and -negative uterine fibroids, respectively, were not significantly enriched with GO terms. The ratio of the number of identified genes to all genes in each term is shown as "geneRatio". P-values were adjusted with the BH method by clusterProfiler^36^ and indicated with colors.

In the MED12m-positive uterine fibroids, three of the 26 COG groups had significant GO terms, while in the MED12m-negative uterine fibroids, five of the 14 COGs groups had significant GO terms (Table 1). Figure 4 shows the specific GO terms from three COG groups in the MED12m-positive (Group1, Group2, and Group3) and five COG groups in the MED12m-negative (Group1, Group2, Group3, Group4, and Group5), respectively. The commonly detected GO terms between the MED12m-positive and -negative uterine fibroids included "RNA splicing, via transesterification reactions", "mRNA splicing, via spliceosome", "ncRNA processing", "mRNA processing", and "RNA splicing", which are related to transcription and translation, and "ribonucleoprotein complex biogenesis" and "DNA replication", which are related to cell proliferation (Fig. 4).

The extracellular matrix-related terms including "extracellular structure organization" and "extracellular matrix organization" were also commonly found. The gene ratios were much larger in the MED12m-positive uterine fibroids than those in the MED12m-negative uterine fibroids, suggesting that the number of extracellular matrix-related genes was larger in the MED12m-positive uterine fibroids than that in the MED12-negative uterine fibroids (Fig. 4).

We then focused on specific GO terms in each of the MED12m-positive or -negative uterine fibroids. There were no specific GO terms to the MED12m-positive uterine fibroids (Fig. 4). On the other hand, the MED12m-negative uterine fibroids had specific GO terms related to cellular protein synthesis ("Golgi vesicle transport", "ER to Golgi vesicle-mediated transport", and "Rho protein signal transduction"), anti-apoptosis ("regulation of apoptotic signaling pathway"), muscles ("muscle system process", "muscle contraction", and "mitochondrial ATP synthesis coupled electron transport"), and angiogenesis ("blood vessel morphogenesis", "angiogenesis", "regulation of vasculature development", and regulation of angiogenesis") (Fig. 4).

These WGCNA results suggest that MED12m-positive uterine fibroids have an increased activity of extracellular matrix organization compared with MED12m-negative uterine fibroids. On the other hand, MED12m-negative uterine fibroids showed increased activities of angiogenesis and smooth muscle cell proliferation. Both types of uterine fibroids had increased activities of cell proliferation and transcription for gene expression.

### DEGs among the three subtypes of MED12m-negative uterine fibroids

Since the hierarchical clustering using DNA methylation profiles classified the MED12m-negative uterine fibroids into the three subtypes as shown in Fig. 1a, it is interesting to know the DEGs among the three subtypes of the MED12m-negative uterine fibroids. Therefore, we compared each subtype of the MED12m-negative uterine fibroids with the normal myometrium. We identified 171 increased and 188 decreased genes in Subtype-1 (Supplementary Table S7 online), 283 increased and 366 decreased genes in Subtype-2 (Supplementary Table S8 online), and 96 increased and 129 decreased genes in Subtype-3 (Supplementary Table S9 online) in comparison with the myometrium. GO enrichment analyses on the increased genes extracted “RNA processing” and “translation” in the Subtypes-1, 2, and 3, suggesting that all subtypes have transcription and protein synthesis activated. This is a common character of the MED12m-positive and MED12m-negative uterine fibroids as shown in Fig. 2. On the other hand, “cell adhesion” and “inflammatory response” were extracted in the decreased genes in all the subtypes (Supplementary Tables S7, S8, and S9 online). Inactivation of cell adhesion is a common character among the three subtypes, and suppression of inflammatory response is a common character of the MED12m-positive and MED12m-negative uterine fibroids as shown in Fig. 2.

The GO terms specific to each subtype were mainly seen in the decreased genes (Supplementary Tables S7, S8, and S9 online). “Wnt signaling pathway” was specific to the subtype-1, and “extracellular matrix organization” and “integrin-mediated signaling pathway” were specific to the subtype-2. In addition, there were a number of other GO terms which are specific to each subtype.

### Immunofluorescence staining

**Figure 5:**
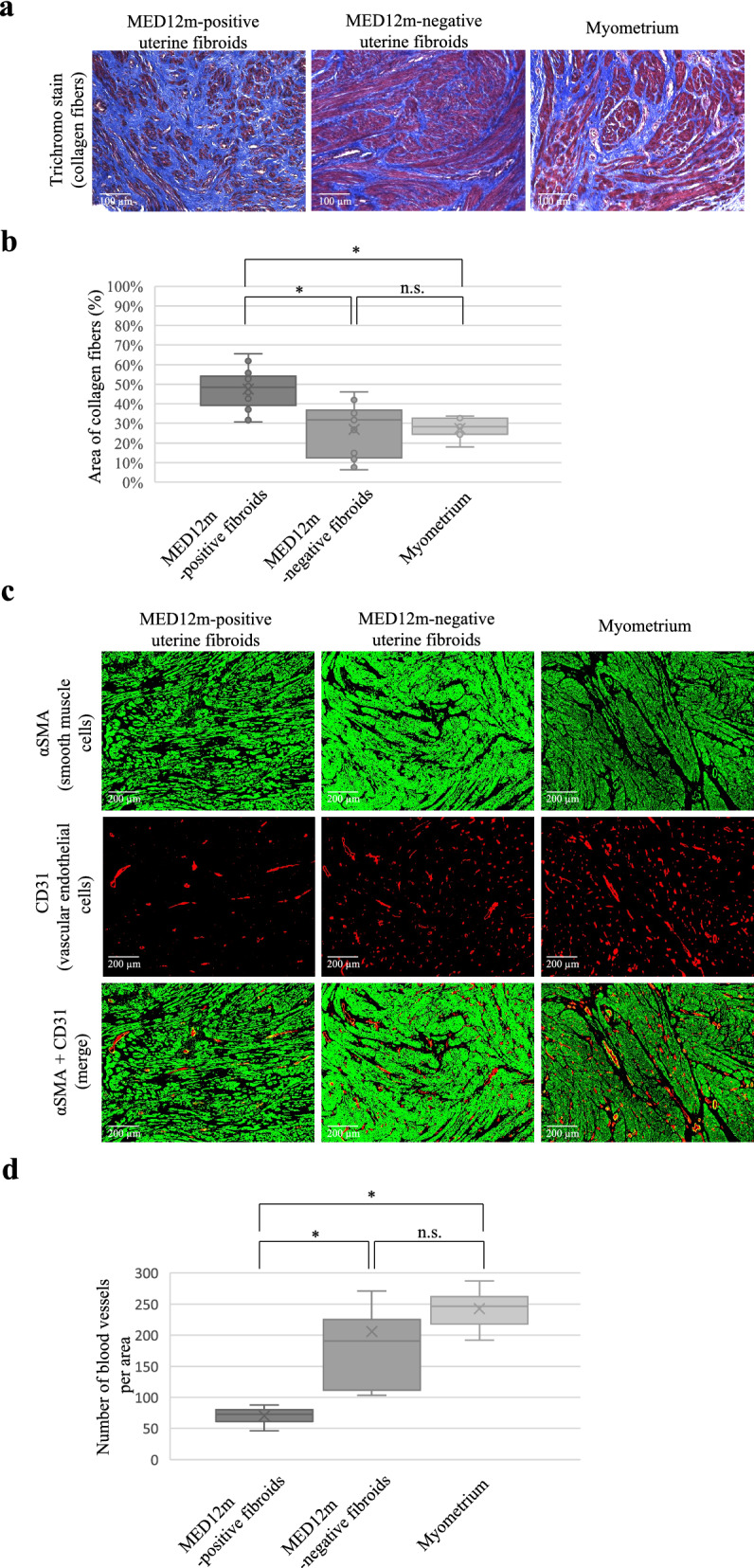

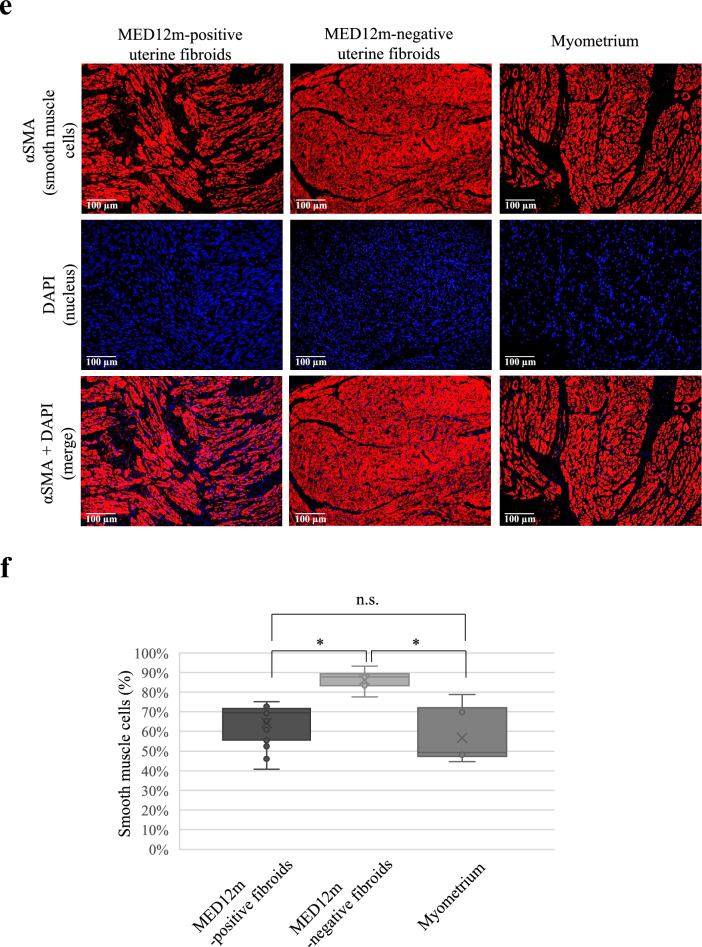
Histological examination in the uterine fibroids and myometrium. (**a**) Immunofluorescent staining for collagen fibers in the MED12m-positive, -negative uterine fibroids, and myometrium. Collagen fibers are detected as blue by a trichrome staining kit (TRM-1, ScyTec Laboratories inc). (**b**) Boxplots show the occupation rate of collagen fiber. The collagen fiber area was quantified by Image J. The percentage per view field was calculated on 15 randomly chosen areas at × 200 magnification, and average percentages were indicated in each tissue section. *p < 0.05. (**c**). Immunofluorescent staining for smooth muscle cells (αSMA, green) and vascular endothelial cells (CD31, red) in the MED12m-positive, -negative uterine fibroids, and myometrium. (**d**). Boxplots show the number of blood vessels, which was counted by Image J. The number per view field was calculated on 5 randomly chosen areas at × 100 magnification, and the average numbers were indicated in each tissue section. *p < 0.05. (**e**) Immunofluorescent staining for smooth muscle cells (αSMA, red) and nucleus (DAPI, blue) in the MED12m-positive, -negative uterine fibroids, and myometrium. The cells stained with αSMA were considered as smooth muscle cells, whereas the cells that were not stained with αSMA was considered as non-smooth muscle cells. (**f**) Boxplots show the percentage of the smooth muscle cells in the smooth and non-smooth muscle cells. The number per view field was calculated on 5 randomly chosen areas at × 200 magnification, and the average numbers were indicated in each tissue section. *p < 0.05.

The GO enrichment analysis in the DEGs and COG groups indicated that the MED12m-positive uterine fibroids have increased activities of extracellular matrix organization and that the MED12m-negative uterine fibroids have increased activities of angiogenesis and proliferation of smooth muscle cells (Figs. 2 and 4). We histologically examined the amount of collagen fibers in the MED12m-positive and -negative uterine fibroids, and myometrium. Immunofluorescence staining showed that the amount of collagen fibers was significantly larger in the MED12m-positive uterine fibroid than in the myometrium and MED12m-negative fibroids (Fig. 5a,b). There was no significant difference between the myometrium and MED12m-negative uterine fibroids. We next examined the number of blood vessels in the MED12m-positive and -negative uterine fibroids, and myometrium. The result showed that the number of blood vessels was significantly higher in the MED12m-negative uterine fibroids than in the MED12m-positive uterine fibroids (Fig. 5c,d). There was no significant difference between the MED12m-negative uterine fibroids and myometrium. We also examined the ratio of smooth muscle cells in total cells in the MED12m-positive, -negative uterine fibroids, and myometrium. The percentage of smooth muscle cells was significantly higher in the MED12m-negative uterine fibroids than the MED12m-positive uterine fibroids and myometrium (Fig. 5e,f). There was no significant difference between the MED12m-positive uterine fibroids and myometrium.

### Upstream regulators in uterine fibroids

**Figure 6:**
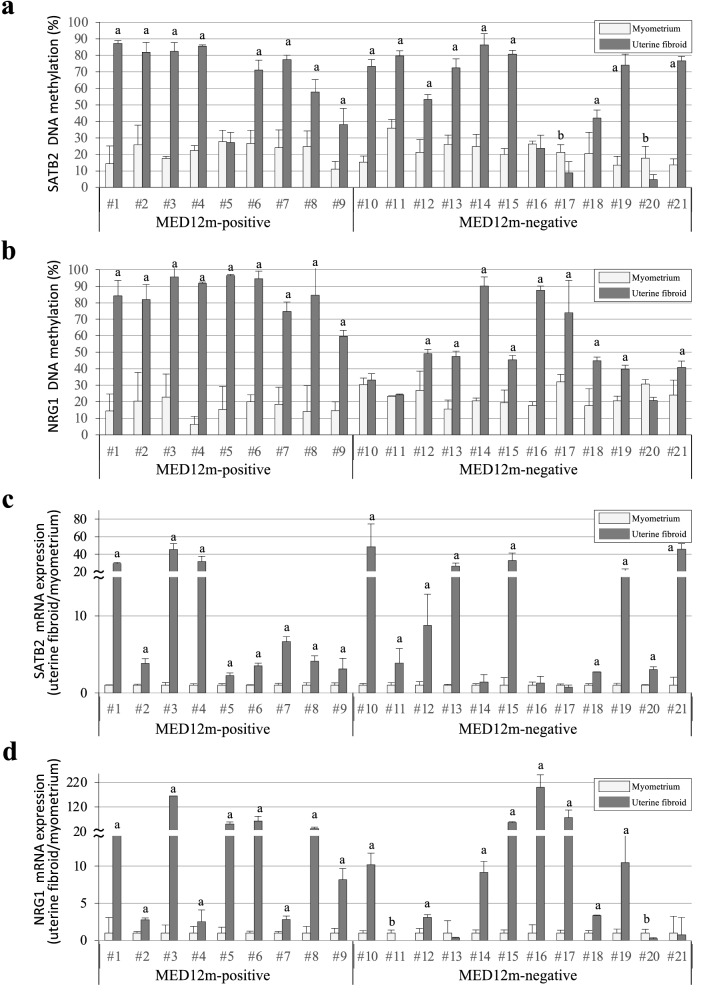
DNA methylation and mRNA expression statuses of SATB2 and NRG1 genes. (**a**,**b**) The DNA methylation levels of SATB2 (**a**) and NRG1 (**b**) genes are shown in dot plots. The vertical axis indicates the DNA methylation levels in the MED12m-positive uterine fibroids (n = 9), -negative uterine fibroids (n = 12), and the corresponding myometrium. The DNA methylation levels were examined by COBRA and range from 0 to 100%. (**c**,**d**) The expression levels of SATB2 (**c**) and NRG1 (**d**) genes in the MED12m-positive uterine fibroids, -negative uterine fibroids, and the myometrium analyzed by qRT-PCR are shown in dot plots. The expression levels are corrected for myometrium expression as 1. ^a^p < 0.01 (higher expression in uterine fibroids compared to myometrium, student t-test). ^b^p < 0.01 (lower expression in uterine fibroids compared to myometrium, student t-test).

To know whether mutations in MED12 are associated with the upregulation of upstream regulators, SATB2 and NRG1 in uterine fibroids, we examined the DNA methylation and expression levels of SATB2 and NRG1 in the uterine fibroids with and without MED12 mutations. In SATB2, 88.9% (8 of 9 samples) of the MED12m-positive uterine fibroids and 75% (9 of 12 samples) of the MED12m-negative uterine fibroids showed higher DNA methylation levels (more than 15% DNA methylation) than the myometrium, respectively (Fig. 6a and Supplementary Table S10 online). In NRG1, 100% (9 of 9 samples) of the MED12m-positive uterine fibroids and 75% (9 of 12 samples) of the MED12m -negative uterine fibroids showed higher DNA methylation levels than the myometrium, respectively (Fig. 6b and Supplementary Table S10 online).

The mRNA expression levels of SATB2 in all nine of the MED12m-positive uterine fibroids and 75% (9 of 12 samples) of the MED12m-negative uterine fibroids were more than twice those in the myometrium (Fig. 6c and Supplementary Table S10 online), while the mRNA expression levels of NRG1 in all the MED12m-positive uterine fibroids and 67% (8 of 12 samples) of the MED12m-negative uterine fibroids were more than twice those in the myometrium, (Fig. 6d and Supplementary Table S10 online). DNA methylation and mRNA expression of at least one of SATB2 and NRG1 were higher in all the MED12m-positive and -negative uterine fibroids than they were in the myometrium. Since DNA hypermethylation and increased expression of SATB2 or NRG1 were observed regardless of MED mutations, these characteristics are unlikely to depend on MED12 mutations.

## Discussion

The present study showed that the DNA methylation profiles of the MED12m-positive and -negative uterine fibroids differed. Since DNA methylation is cell/tissue-specific, uterine fibroids with MED12 mutations differ from uterine fibroids without MED12 mutations at the molecular level. This prompted us to clarify the difference between the two types of uterine fibroids in this study.

In the MED12m-positive uterine fibroids, DEGs were enriched to the GO terms related to extracellular matrix organization. The WGCNA analysis also showed the activated extracellular matrix organization in the MED12m-positive uterine fibroids. On the other hand, in the MED12m-negative uterine fibroids, most of the genes in the GO terms related to extracellular matrix organization were down-regulated in comparison with the myometrium and MED12m-positive uterine fibroids. Furthermore, the TGF-beta signaling pathway, which contributes to fibrosis^22,23^, was downregulated in the MED12m-negative uterine fibroids. In addition, GO analysis with the genes whose promoters are differentially methylated between MED12m-positive and MED12m-negative uterine fibroids also suggests that TGF-beta signaling and extracellular matrix organization are more activated in the MED12m-positive uterine fibroids compared with the MED12m-negative uterine fibroids. These results suggest that MED12 mutations activate extracellular matrix organization in uterine fibroids. In fact, our histological results showed that the amount of collagen was enriched in the uterine fibroids with MED12 mutations. Many reports have shown the increased expression of COL4A1 and COL4A2, which contribute to collagen synthesis, and increased collagen deposition in uterine fibroids^11,24^. Also, many of the uterine fibroids in those studies may have been MED12m-positive because more than 70% of uterine fibroids are MED12m-positive.

The WGCNA analysis in the MED12m-negative uterine fibroids detected a COGs group that is related to muscles. In our histological results, the amount of smooth muscle cells was larger in the uterine fibroids without MED12 mutations compared with the uterine fibroids with MED12 mutations, which is consistent with a previous report demonstrating a high ratio of smooth muscle cells to fibroblasts in the uterine fibroids without MED12 mutations compared to that with MED12 mutations^9^. That study, together with the present results suggest that uterine fibroids without MED12 mutations are enriched in smooth muscle cells and contain a low amount of collagen fibers, and that MED12 mutations are associated with collagen-rich uterine fibroids.

The GO enrichment analyses with DEGs and WGCNA analysis showed that both MED12m-positive and -negative uterine fibroids have increased cell proliferation and transcription activities. This well reflects one of the characters of uterine fibroids, which is the activated cell proliferation of smooth muscle cells or fibroblasts. It is interesting to note that the activity of the Wnt signaling pathway was decreased in MED12m-negative uterine fibroids. The Wnt signaling pathway has been reported to play an important role in the growth of uterine fibroids^22^. This may be because the major type of uterine fibroids included in those reports was MED12m-positive uterine fibroids in which Wnt signaling pathway is activated. We speculate that the growth of MED12m-negative uterine fibroids is regulated by signaling pathways other than the Wnt signaling pathway. As shown in Table 2, multiple signaling pathways are involved in cell proliferation in both MED12m-positive and -negative uterine fibroids.

**Table 2:** Activated signalings of cell proliferation and anti-apoptosis in differentially expressed genes in uterine fibroids.

IPA pathway (p < 0.05)	MED12m-positive	MED12m-negative
Cell proliferation		
**Wnt/β-catenin signaling**	●	
HIF1α- signaling	●	
mTOR signaling	●	●
PI3K/AKT signaling	●	●
p70S6K signaling	●	●
STAT3 siganling	●	●
RANK signaling in osteoclasts	●	
PPARα/RXTα activation	●	
NF-κB signaling	●	
RAR activation	●	●
PXR/RXR activation		●
HER-2/ErbB signaling		●
ERK/MAPK siganling		●
LPS-stimulated MAPK signaling		●
**Anti-apoptosis**		
14–3-3mediated signaling	●	
PI3K/AKT signaling	●	●
p70S6K signaling	●	●
PAK signaling		●
IGF-1 signaling		●

Differentially expressed genes compared to the myometrium (the DEGs) in the MED12m-positive and -negative uterine fibroids were applied to KEGG pathway analysis in IPA, respectively. Detected pathways with p < 0.05 were considered significant enrichment. Activated signaling pathways related to cell proliferation and anti-apoptosis were indicated. Differentially expressed genes compared to the myometrium (the DEGs) in the MED12m-positive and -negative uterine fibroids were applied to KEGG pathway analysis in IPA, respectively. Detected pathways with p < 0.05 were considered significant enrichment. Activated signaling pathways related to cell proliferation and anti-apoptosis were indicated.

VEGF expression is reported to be upregulated in uterine fibroids^25^, which suggests that angiogenic activity in increased in uterine fibroids. On the other hand, our GO enrichment analysis suggested that angiogenesis is downregulated in MED12m-positive uterine fibroids and upregulated in MED12m-negative uterine fibroids. There seems to be a discrepancy between the previous reports and our results. That may be due to the difference in cellular components of the tissue samples of uterine fibroids. As shown in the histological features of both types of uterine fibroids, MED12m-positive uterine fibroids are collagen-rich while MED12m-negative uterine fibroids are cell-rich. Collagen-rich tissues should show low angiogenesis while cell-rich tissues should show high angiogenic activity. In fact, our result indicated that the MED12m-negative uterine fibroids had higher number of blood vessels compared with the MED12m-positive uterine fibroids.

In addition, the response to estrogen was found to be upregulated in MED12m-positive uterine fibroids. Since fibroblasts were reported to proliferate or produce collagen in response to estrogen while smooth muscle cells proliferate in response to progesterone^9^, collagen-rich MED12m-positive uterine fibroids seem to well respond to estrogen.

Our results also suggested that the immune response and reactive oxygen species metabolic processes are decreased in both MED12m-positive and -negative uterine fibroids. That is not surprising because tumorigenesis is well known to occur under the suppressive environment of immune responses and reactive oxygen species^25–28^.

High mobility group AT-hook2 (HMGA2) mutation is considered to be one of the mutations driving the development of uterine fibroids^3,29,30^, and the MED12 mutations and rearrangement of HMGA2 have been shown to occur in a mutually exclusive manner^4,5^. However, our results showed that some uterine fibroids carried both the MED12 mutation and HMGA2 overexpression, and that only half of the MED12m-negative fibroids had increased HMGA2 expression (Supplementary Fig. S2 online). Previous reports also indicated that the MED12 mutations and increased HMGA2 expression co-existed in the same uterine fibroid nodule^10,31^.

We previously reported that SATB2 and NRG1 act as upstream regulatory factors in the pathogenesis of uterine fibroids^19,22,23^. Whether or not MED12 has a mutation, SATB2 and NRG1 were more strongly expressed in uterine fibroids than in the myometrium, which indicates that the upregulation of NRG1 and SATB2 are independent of MED12 mutations. Our results also indicated that all the MED12m-positive and -negative uterine fibroids had DNA hypermethylation and increased mRNA expression in either SATB2 or NRG1, suggesting that the dysregulation of upstream regulatory factors such as SATB2 and NRG1 is involved in the pathogenesis of uterine fibroids.

One may question the relationship between MED12 mutation and DNA methylation; whether MED 12 mutation changes DNA methylation status. It is unlikely that a MED 12 mutation could change the DNA methylation status because the DNA methylation profile of Subtype-1 MED12m-negative uterine fibroids was identical to the MED12m-positive uterine fibroids. Further studies are needed to identify the differences among the three subtypes of uterine fibroids without MED12 mutation.

The hierarchical clustering using DNA methylation profiles indicated the possibility that MED12m-negative uterine fibroids can be classified into the three subtypes. DNA methylation on the other regions than the promoters may be rather associated with subtyping in the MED12m-negative uterine fibroids. It is also interesting to know whether there is a character specific to each subtype in the MED12m-negative uterine fibroids, GO analysis with DEGs among the three subtypes suggested that inactivation of Wnt signaling pathway is specific to the subtype-1, and decreased activities of extracellular matrix organization and integrin-mediated signaling pathway are specific to the subtype-2, while activation of transcription and protein synthesis, and inactivation of cell adhesion and inflammatory response are common in the three subtypes. Our results strongly suggest that there are three subtypes in the MED12m-negative uterine fibroids in which each subtype has several specific characters. However, since the number of samples of each subtype was limited (3 samples each), further studies with more samples are needed whether the MED12m-negative uterine fibroids can be classified into multiple subtypes.

In conclusion, the present study shows that uterine fibroids with and without MED12 mutations clearly differ in DNA methylation, gene expression, and histological features. The DNA methylome indicated that the uterine fibroids carrying MED12 mutations differed from the uterine fibroids without MED12 mutations, and that MED12 mutations do not directly change DNA methylation profiles of uterine fibroids. The transcriptome and histological examination revealed that the MED12m-positive uterine fibroids increased extracellular matrix production activity compared with the MED12m-negative uterine fibroids. MED12 mutations may affect the phenotypes of uterine fibroids by modulating the production of extracellular matrix. Both types of uterine fibroids had increased cell proliferation activities, but they may use different signaling pathways for growth. The present study shows that uterine fibroids differ depending on the presence of MED12 mutations.

## Methods

### Ethics statement

This study was reviewed and approved by the Institutional Review Board of Yamaguchi University Graduate School of Medicine. Written informed consent was obtained from the participants before collecting any samples, and the specimens were irreversibly de-identified. All experiments involving the handling of human tissues were performed following the Tenets of the Declaration of Helsinki.

### Tissue preparation

Tissues of uterine fibroid and myometrium were obtained from 42 Japanese women, respectively. Uterine fibroids were obtained from patients aged 33–45 who underwent hysterectomy for uterine fibroids. Myometrium was obtained from patients with uterine fibroids aged 34–42 who underwent hysterectomy for uterine fibroids or early stage of cervical cancer. None of the women enrolled in this study received previous treatment with sex steroid hormones or gonadotropin-releasing hormone agonists/antagonists. We analyzed the uterine fibroids with MED12 mutation status by Sanger sequencing as reported previously (Supplementary Fig. S1 online)^16^. All of the MED12m-positive uterine fibroids had the point mutations in MED12 gene. MED12 expression levels were not significantly different between myometrium and uterine fibroids with or without MED12 mutation.

### HMGA2 expression statuses

HMGA2 rearrangements are thought to be one of the mutations driving the development of uterine fibroids^3,30^. Among the uterine fibroids without MED12 mutations, uterine fibroids carrying HMGA2 rearrangements oocur with the highest frequency^3,30^. More than 80% of uterine fibroids possess karyotypic abnormalities, and MED12 mutations and HMGA2 rearrangements encompass approximately 80–90% of genetic alterations in uterine fibroids^3,29,30^. Previous reports indicated that the MED12 mutations and rearrangement of HMGA2 occur in a mutually exclusive manner^4,5^. Therefore, it has been suggested that the MED12 mutations and HMGA2 rearrangements were alternatively associated with the pathogenesis of uterine fibroids. Hence, past reports compared the features between the uterine fibroids with the MED12 mutations and that with HMGA2 rearrangements^9,10^. The uterine fibroids carrying HMGA2 rearrangement are reported to overexpress HMGA2^10^. To investigate whether our samples included the uterine fibroids carrying HMGA2 rearrangement, we examined the expression levels of HMGA2 in the MED12m-negative and MED12m-positive uterine fibroids, and myometrium using transcriptome analyses. Three of the nine MED12m-negative uterine fibroids had expressions more than two-fold of the mean expression in the myometrium (Supplementary Fig. S2 online). Moreover, one of the six MED12m-positive uterine fibroids had expressions more than two-fold of the mean expression in the myometrium (Supplementary Fig. S2 online). These results suggest that (1) a number of the MED12m-negative uterine fibroids lack HMGA2 rearrangements, and (2) MED12 mutations and HMGA2 rearrangements can co-exist. These facts led us to compare the uterine fibroids with and without MED12 mutations rather than to compare uterine fibroids with MED12 mutations and HMGA2 rearrangements.

### Illumina Infinium HumanMethylation450 BeadChip Assay

Genomic DNA was isolated from the uterine fibroids and myometrium using a Qiagen Genomic DNA kit (Qiagen, Valencia, CA, USA), as previously reported^32^. DNA methylation was analyzed with an Illumina Infinium assay with the HumanMethylation450 BeadChip (Illumina, San Diego, CA, USA), which interrogates a total of 482,421 CpGs spread across the distal promoter regions of the transcription start sites to 3’-UTR of consensus coding sequences. Methylated and unmethylated signals were used to compute beta-values, which are quantitative scores of the DNA methylation levels, ranging from 0 (completely unmethylated) to 1 (completely methylated). The BeadChip was scanned on a BeadArray Reader (Illumina) according to the manufacturer's instructions. CpGs with "detection p values" > 0.01 (computed from the background based on negative controls), CpGs that were zero in all samples, and CpGs on Y chromosome were eliminated from further analysis, leaving 422,165 CpGs valid for use. The DNA methylation data of the CpGs were normalized in genome studio. We used NCBI Reference Sequence Database (https://www.ncbi.nlm.nih.gov/refseq/) as reference genes.

### Transcriptome analysis

The transcriptomes of myometrium, MED12m-positive, and -negative uterine fibroids were analyzed as previously reported^33,34^. Total RNAs were isolated from cells by using an RNeasy mini kit (Qiagen). Target cDNA for a microarray was prepared from 250 ng of total RNA with the Ambion WT Expression kit (Ambion, Austin, TX, USA) and the GeneChip WT PLUS reagent kit (Affymetrix). Transcriptomes were analyzed with a GeneChip Human Genome 1.0 ST Array (Affymetrix, Santa Clara, CA, USA) as previously reported^33,34^. The microarray was spotted with 21,014 RefSeq genes. Hybridization to the microarrays, washing, staining, and scanning was performed using the GeneChip system (Affymetrix) composed of the Scanner 30,007 G Workstation Fluidics 450 and the Hybridization Oven 645. The scanned image data were processed using a gene expression analysis with the Patrek Genomics Suite 6.5 software program (Partech, Munster, Germany). All expression data were converted to log2 values. Differentially expressed genes (DEGs) were extracted when the expressions in the MED12m-positive or -negative uterine fibroids were higher than 2.0-fold or less than 0.5-fold of that in the myometrium, and p < 0.05 (t-test), and the average expression levels in the tissues with higher expression were more than 100.

### Weighted gene co-expression network analysis (WGCNA)

We employed a weighted gene co-expression network analysis (WGCNA) package in R^20,21^ according to the manufacturer's instructions to identify genes that were co-expressed in uterine fibroids and myometrium. Low signal probes with values < 10 in more than 90% of the samples were considered as noise and removed, and correlations based on mostly zero counts are not meaningful. To stabilize the samples' variance, we used the variance stabilizing transformation function in DESeq2^35^. The blockwise-Module function was used with the parameters; power = 12; minimum module size = 50; deep split = 0; cut height = 0.95; gene group merge height = 0.25. Detected gene groups were subjected to gene annotation analysis using clusterProfiler in R^36^. The ratio of the number of identified genes to all genes in each term was calculated. P-values were adjusted by the Benjamini and Hochberg (BH) method that is the default p-value adjustment in clusterProfiler^36^.

### Immunohistochemistry

Collagen fibers were stained and visualized in tissue sections using the Trichrome Stain Kit (TRM-1, ScyTec Laboratories inc., Utah, USA) following the manufacturer's instructions. Tissue sections (5 µm) of paraffin-embedded samples were deparaffinized, washed with cold phosphate-buffered saline (PBS), placed in preheated Bouin's fluid overnight, rinsed in tap water until completely clear, rinsed in distilled water, stained with Weigert's Iron Hematoxylin for 10 min, rinsed in tap water for 2 min, rinsed in distilled water, immersed in Biebrich Scarlet/Acid Fuchsin Solution for 10 min, rinsed in distilled water, defferentiated in Phosphomolybdic/Phosphotungstic Acid Solution for 15 min, placed in Aniline Blue Solution for 15 min, rinsed in distilled water, immersed in Acetic Acid Solution (1%) for 1 min, dried, placed in Xylene 3 times and mounted in synthetic resin. The area of collagen fibers, which were stained blue, was quantified by Image J, and the percentage per field of view was calculated. The calculations were done on 15 randomly chosen areas at × 200, and the average percentages were used as the collagen fiber area (%) in each tissue.

Tissue sections (5 µm) of paraffin-embedded samples were deparaffinized, washed with cold phosphate-buffered saline (PBS), and blocked with blocking solution (10% bovine fetal serum and 1% bovine serum albumin in PBST) for 60 min. Then the cells were incubated with mouse anti-αSMA monoclonal antibody for smooth muscle cell staining (Abcam, Tokyo, Japan; Cat# ab7817, RRID: AB_262054) and rabbit anti-CD31 monoclonal antibody for vascular endothelial cells (Abcam, Cat# ab182981, RRID: AB_2756834) as primary antibody (diluted at 1:500 in the blocking solution) at 4 C overnight, and incubated with the Alexa Fluor 488 or 594 conjugated goat anti-mouse IgG (Abcam, Cat# ab150113: RRID: AB_2576208; Abcam, Cat# ab150116: RRID: AB_2650601) and the Alexa Fluor 594 conjugated goat anti-rabbit IgG (Abcam, Cat# ab150084, RRID: AB_2734147) as secondary antibodies (diluted at 1:1000 in PBS) for 45 min a room temperature, respectively. The number of blood vessels, which were stained red larger than 15 pixels, was counted by Image J, and the number per field of view was calculated. The calculations were done on 5 randomly chosen areas at × 100, and the average numbers were used in each tissue. The percentages of smooth muscle cells were calculated as follows: the number of smooth muscle cells/the number of smooth and non-smooth muscle cells. Cells stained with or without αSMA were considered as smooth muscle cells or non-smooth muscle cells. The number per view field was calculated on 5 randomly chosen areas at × 200 magnification, and the average numbers were indicated in each tissue section.

The amounts of collagen fibers, the number of blood vessels, and the percentage of smooth muscle cells between the MED12m-positive and -negative uterine fibroids and myometrium were compared with pairwise Wilcoxon rank-sum tests using R (function "pairwise.wilcox.test"; version 3.6.0.). p < 0.05 was considered significant.

### Bioinformatics

DAVID Bioinformatics Resources v. 6.8 (https://david.ncifcrf.gov/) and Ingenuity Pathway Analysis (IPA, Qiagen) were used to determine whether the functional annotation of the differentially expressed genes was enriched for specific Gene Ontology (GO) terms and biological pathways, respectively^37^. In GO analysis, GO terms with adjusted p (BH method) < 0.01 were considered significant enrichment. In the pathway analysis, pathways with p < 0.05 were considered significant enrichment. In WGCNA analysis, adjusted p < 0.1 was regarded as substantial enrichment in the GO enrichment analysis. Hierarchical clustering was performed in R using the Ward method^38^. Chromosomal distributions of the DNA methylation statuses of all CpG loci in the MED12m-positive and -negative uterine fibroids compared to the myometrium were examined using "chromoMap" implemented in R (https://cran.r-project.org/web/packages/chromoMap/index.html). CpG sites, which have p < 0.05 and beta-value difference > 0.2 compared to the myometrium, are plotted in autosomes and X chromosome. The GO terms were summarized by removing redundancy and plotted using reduce and visualize gene ontology (REVIGO) with Allowed Similarity as "small (0.5)"^39^.

### Combined bisulfite restriction analysis (COBRA)

DNA methylation levels were evaluated by COBRA as we previously reported^15,16^. In brief, sodium bisulfite treatment was performed using an EpiTect Bisulfite kit (Qiagen) according to the conditions as follows: 95 °C for 5 min, 65 °C for 85 min, 95 °C for 5 min and 65 °C for 175 min. After sodium bisulfite treatment, PCR was performed using one unit of Biotaq HS DNA polymerase (Bioline, London, UK) and the primer sets shown in Supplementary Table S11 online under the thermocycling conditions (35 to 38 cycles of 95 °C for 30 s, 60 °C for 30 s, and 72 °C for 30 s, with an initial step of 95 °C for 10 min and a final step of 72 °C for 7 min). A part of the PCR product was digested with the restriction enzyme TaqI (Takara, Tokyo, Japan) or HpyCH4IV (New England Biolabs, Ipswich, MA). The treated PCR product was electrophoresed by 3% agarose gel. PCR products from methylated DNA and unmethylated DNA are digested and undigested by the treatment with the restriction enzyme. The intensity of the signals of the digested and undigested PCR products was measured by densitometry. Methylation levels (%) were calculated as the ratio of the digested PCR product in the total PCR product (digested + undigested products).

### Quantitative real-time RT-PCR (qRT-PCR)

Total RNA was isolated from tissues and cells using Isogen (Wako Pure Chemical Industries Ltd, Osaka, Japan). One µg total RNA was reverse-transcribed using a Quantitect Reverse Transcription Kit (Qiagen) according to the manufacturer's protocol as previously reported^15^. A primer pair for glyceraldehyde-3-phosphate dehydrogenase (*GAPDH*) was used as an internal control. Real-time qRT-PCR was performed using SYBR Premix Ex Taq (Takara, Ohtsu, Japan) and a LightCycler (Roche Applied Science, Basel, Switzerland). All samples were run in duplicate. The relative quantity of cDNA was calculated with the ∆∆Ct method. Melting curves of the products were obtained after cycling by a stepwise increase of temperature from 55 to 95 °C. The primer sequences used in this analysis are shown in Supplementary Table S11 online.

### Statistical analysis

All statistical analyses were performed in R^38^. The DNA methylation and expression levels between the myometrium and the MED12m-positive and -negative uterine fibroids were compared with student t-tests using R (function "t.test"; version 3.6.0.). p < 0.05 was considered significant.

### Computing platform

The computing platform used in this study was an Intel(R) Xeon(R) CPU E5-2667 v4, 3.20 GHz (× 4 CPUs, eight cores per CPU) with 504 GB RAM running CentOS release 6.10.

## Supplementary Information

Supplementary Figure S1.

Supplementary Figure S2.

Supplementary Table S1.

Supplementary Table S2.

Supplementary Table S3.

Supplementary Table S4.

Supplementary Table S5.

Supplementary Table S6.

Supplementary Table S7.

Supplementary Table S8.

Supplementary Table S9.

Supplementary Table S10.

Supplementary Table 11.

## Data Availability

The data underlying this article are available in the Dryad Digital Repository at https://doi.org/10.5061/dryad.sn02v6x4d.

## References

[1] Stewart EA (2001). Uterine fibroids. Lancet.

[2] Makinen N (2011). MED12, the mediator complex subunit 12 gene, is mutated at high frequency in uterine leiomyomas. Science.

[3] Mehine M (2013). Characterization of uterine leiomyomas by whole-genome sequencing. N. Engl. J. Med..

[4] Bullerdiek J, Rommel B (2018). Factors targeting MED12 to drive tumorigenesis?. F1000Res.

[5] Markowski DN (2012). MED12 mutations in uterine fibroids–their relationship to cytogenetic subgroups. Int. J. Cancer.

[6] Mittal P (2015). Med12 gain-of-function mutation causes leiomyomas and genomic instability. J. Clin. Invest..

[7] Asano R (2015). Aberrant expression of erythropoietin in uterine leiomyoma: Implications in tumor growth. Am. J. Obstet. Gynecol..

[8] Asano R (2019). Expression of erythropoietin messenger ribonucleic acid in wild-type MED12 uterine leiomyomas under estrogenic influence: New insights into related growth disparities. Fertil. Steril..

[9] Wu X (2017). Subtype-specific tumor-associated fibroblasts contribute to the pathogenesis of uterine leiomyoma. Cancer Res..

[10] George JW (2019). Integrated epigenome, exome, and transcriptome analyses reveal molecular subtypes and homeotic transformation in uterine fibroids. Cell Rep..

[11] Liu X, Liu Y, Zhao J, Liu Y (2018). Screening of potential biomarkers in uterine leiomyomas disease via gene expression profiling analysis. Mol. Med. Rep..

[12] Kim M, Costello J (2017). DNA methylation: An epigenetic mark of cellular memory. Exp. Mol. Med..

[13] Maekawa R (2019). Aberrant DNA methylation suppresses expression of estrogen receptor 1 (ESR1) in ovarian endometrioma. J. Ovarian Res..

[14] Maekawa R (2016). Tissue-specific expression of estrogen receptor 1 is regulated by DNA methylation in a T-DMR. Mol. Endocrinol..

[15] Maekawa R (2013). Genome-wide DNA methylation analysis reveals a potential mechanism for the pathogenesis and development of uterine leiomyomas. PLoS ONE.

[16] Sato S (2016). Identification of uterine leiomyoma-specific marker genes based on DNA methylation and their clinical application. Sci. Rep..

[17] Maekawa R (2011). Disease-dependent differently methylated regions (D-DMRs) of DNA are enriched on the X chromosome in uterine leiomyoma. J. Reprod. Dev..

[18] Sato S (2014). Potential mechanisms of aberrant DNA hypomethylation on the x chromosome in uterine leiomyomas. J. Reprod. Dev..

[19] Sato S (2019). SATB2 and NGR1: Potential upstream regulatory factors in uterine leiomyomas. J. Assist. Reprod. Genet..

[20] Zhang B, Horvath S (2005). A general framework for weighted gene co-expression network analysis. Stat. Appl. Genet. Mol. Biol..

[21] Langfelder P, Horvath S (2008). WGCNA: An R package for weighted correlation network analysis. BMC Bioinform..

[22] Borahay MA, Al-Hendy A, Kilic GS, Boehning D (2015). Signaling pathways in leiomyoma: Understanding pathobiology and implications for therapy. Mol. Med..

[23] Ciebiera M (2017). Role of transforming growth factor beta in uterine fibroid biology. Int. J. Mol. Sci..

[24] Reis FM, Bloise E, Ortiga-Carvalho TM (2016). Hormones and pathogenesis of uterine fibroids. Best Pract. Res. Clin. Obstet. Gynaecol..

[25] Zannotti A (2021). Macrophages and immune responses in uterine fibroids. Cells.

[26] Leppert PC, Catherino WH, Segars JH (2006). A new hypothesis about the origin of uterine fibroids based on gene expression profiling with microarrays. Am. J. Obstet. Gynecol..

[27] Orciani M (2018). Chronic inflammation may enhance leiomyoma development by the involvement of progenitor cells. Stem Cells Int..

[28] Fletcher NM (2013). Uterine fibroids are characterized by an impaired antioxidant cellular system: Potential role of hypoxia in the pathophysiology of uterine fibroids. J. Assist. Reprod. Genet..

[29] Bertsch E (2014). MED12 and HMGA2 mutations: Two independent genetic events in uterine leiomyoma and leiomyosarcoma. Mod. Pathol..

[30] Mehine M, Makinen N, Heinonen HR, Aaltonen LA, Vahteristo P (2014). Genomics of uterine leiomyomas: Insights from high-throughput sequencing. Fertil. Steril..

[31] Mello JBH (2018). MicroRNAs involved in the HMGA2 deregulation and its co-occurrence with MED12 mutation in uterine leiomyoma. Mol. Hum. Reprod..

[32] Maekawa R (2019). Genome-wide DNA methylation analysis revealed stable DNA methylation status during decidualization in human endometrial stromal cells. BMC Genom..

[33] Yamagata Y (2014). Genome-wide DNA methylation profiling in cultured eutopic and ectopic endometrial stromal cells. PLoS ONE.

[34] Mihara Y (2020). An integrated genomic approach identifies HOXC8 as an upstream regulator in ovarian endometrioma. J. Clin. Endocrinol. Metab..

[35] Love MI, Huber W, Anders S (2014). Moderated estimation of fold change and dispersion for RNA-seq data with DESeq2. Genome Biol..

[36] Yu G, Wang LG, Han Y, He QY (2012). clusterProfiler: An R package for comparing biological themes among gene clusters. OMICS.

[37] da Huang W (2007). DAVID Bioinformatics Resources: Expanded annotation database and novel algorithms to better extract biology from large gene lists. Nucleic Acids Res..

[38] R Core Team (2021). R: A Language and Environment for Statistical Computing.

[39] Supek F, Bosnjak M, Skunca N, Smuc T (2011). REVIGO summarizes and visualizes long lists of gene ontology terms. PLoS ONE.

