# Supplementary material for: Different DNA methylome, transcriptome and histological features in uterine fibroids with and without MED12 mutations
Source: Sci Rep. 2022 May 26;12:8912. doi: 10.1038/s41598-022-12899-7 (PMC9135739; doi:10.1038/s41598-022-12899-7)
Supplement: Supplementary file 3 — Supplementary Table S1. [file 41598_2022_12899_MOESM3_ESM.pdf]

Different DNA methylome, transcriptome and histological features in uterine fibroids with and without MED12 mutations

Ryo Maekawa*, Department of Obstetrics and Gynecology, Yamaguchi University Graduate School of Medicine, Ube, 755-8505 Japan

Shun Sato, Department of Obstetrics and Gynecology, Yamaguchi University Graduate School of Medicine, Ube, 755-8505 Japan

Tetsuro Tamehisa, Department of Obstetrics and Gynecology, Yamaguchi University Graduate School of Medicine, Ube, 755-8505 Japan

Takahiro Sakai, Department of Obstetrics and Gynecology, Yamaguchi University Graduate School of Medicine, Ube, 755-8505 Japan

Takuya Kajimura, Department of Obstetrics and Gynecology, Yamaguchi University Graduate School of Medicine, Ube, 755-8505 Japan

Kotaro Sueoka, Department of Obstetrics and Gynecology, Yamaguchi University Graduate School of Medicine, Ube, 755-8505 Japan

Norihiro Sugino, Department of Obstetrics and Gynecology, Yamaguchi University Graduate School of Medicine, Ube, 755-8505 Japan

Supplemental Table S1. Increased 157 genes in the MED12m-positive uterine fibroids compared to the myometrium.

Gene symbol	Myometrium (mean log2 value)	MED12m-positive (mean log2 value)	pvalue	fold change (log2)
ADAM12	5.422913333	9.39171	0.04759629	3.968796667
ANKRD20A11P	4.688646667	6.947313333	0.000834011	2.258666667
APOE	8.436206667	9.7836	0.01988447	1.347393333
ATP8B4	7.517466667	9.22794	0.007370679	1.710473333
C1QA	6.42138	7.726036667	0.009037761	1.304656667
C1QB	7.89037	9.316896667	0.042026086	1.426526667
CAMK1G	5.945663333	7.359053333	0.044382621	1.41339
CAPN6	8.36719	12.49338667	0.008123616	4.126196667
CCND1	8.468586667	10.52389	0.021189081	2.055303333
CD24	8.62464	10.50618333	0.035027281	1.881543333
CDH8	5.59772	8.076816667	0.027585518	2.479096667
CKS2	5.67796	8.69129	0.023914641	3.01333
CNTN4	7.22025	8.30322	0.017360505	1.08297
COL4A1	9.722363333	11.12408667	0.012538642	1.401723333
COL4A2	9.482096667	10.87648333	0.00768962	1.394386667
CRABP2	7.859966667	9.698476667	0.006139782	1.83851
DCX	5.438426667	10.79778667	0.024635272	5.35936
DKK2	6.004376667	7.91324	0.001167357	1.908863333
DNAH14	5.40168	7.638176667	0.049408205	2.236496667
EDA2R	7.431523333	9.339376667	0.010020163	1.907853333
EGFL6	5.123846667	8.127366667	0.039283196	3.00352
ELFN1	6.801236667	8.044706667	0.032722229	1.24347
F2RL2	7.421633333	9.074083333	0.047956583	1.65245
FAM212B	5.981703333	7.38469	0.021012869	1.402986667
FAM229B	7.626286667	9.124636667	0.009767166	1.49835
FCGR1C	6.46266	8.621743333	0.047905324	2.159083333
FCGR2A	7.177956667	8.585713333	0.039678814	1.407756667
FCGR3B	6.69853	8.984753333	0.030465242	2.286223333
FHOD3	5.785456667	6.919053333	0.035043074	1.133596667
GALNT13	5.601096667	8.85739	0.00450621	3.256293333
GGH	5.786576667	7.11289	0.005878918	1.326313333
GRIN2A	5.58568	8.40352	0.034107575	2.81784
H2AFZ	9.062193333	10.15833667	0.007305764	1.096143333
HIST1H1D	7.30653	8.536256667	0.012042906	1.229726667
HIST1H2BG	5.678336667	6.709666667	0.025236415	1.03133
HIST1H2BH	6.769416667	8.309353333	0.039565312	1.539936667
HIST1H4C	8.927526667	10.68711	0.002178193	1.759583333
HIST1H4F	7.090936667	8.761933333	0.029598891	1.670996667
HIST1H4J	8.189606667	10.52002333	0.022202703	2.330416667
HIST1H4K	8.207853333	10.78962667	0.011577612	2.581773333
IGSF3	5.902806667	8.913696667	0.013052975	3.01089
INS	6.946963333	8.083373333	0.001866846	1.13641
ITM2C	8.955956667	10.59957333	0.003957503	1.643616667
KIF5C	6.070986667	8.98742	0.020159935	2.916433333
LOC101060256	5.633333333	6.764363333	0.005107041	1.13103
LOC102723462	5.375436667	7.15014	0.001647902	1.774703333
LRRC7	4.799883333	9.53976	0.000596906	4.739876667
LRRN1	5.16803	7.24897	0.045102584	2.08094
LRRTM1	5.33428	6.771566667	0.048634723	1.437286667
MEST	8.246683333	10.23329667	0.027865713	1.986613333
MEX3B	6.69355	7.92038	0.001868311	1.22683
MFAP2	7.904486667	9.831586667	0.028408679	1.9271
MMP16	6.732336667	8.55153	0.009587935	1.819193333
MSR1	6.793213333	7.907763333	0.042869296	1.11455
MTCL1	6.2671	7.74076	0.02667011	1.47366
NAV2	8.13147	9.203343333	0.04391314	1.071873333
NDUFA6	8.085033333	9.127296667	0.017270889	1.042263333
NETO2	6.020346667	8.574523333	0.007439846	2.554176667
NHSL2	6.74297	7.821293333	0.016989287	1.078323333
NNAT	6.277476667	7.9811	0.006417599	1.703623333
NPTX2	6.235363333	7.343733333	0.026631293	1.10837

NTNG1	5.188786667	7.053853333	0.006325969	1.865066667
OLFM1	7.121226667	8.158416667	0.026161139	1.03719
PAGE4	4.22231	7.10244	0.004309832	2.88013
PARP9	8.48129	9.635393333	0.006163065	1.154103333
PCDH10	5.84376	8.02948	0.000233046	2.18572
PCP4	10.3973	12.34629667	0.032111244	1.948996667
PHGDH	7.028563333	8.172746667	0.004061869	1.144183333
PLEKHG4B	5.701913333	6.976753333	0.002374067	1.27484
POLH	7.255463333	8.365703333	0.025063259	1.11024
PRLHR	5.951256667	7.46183	0.010221077	1.510573333
PTCHD4	7.070283333	9.7155	0.016537881	2.645216667
RNU11	8.97137	10.23241333	0.006033104	1.261043333
RNVU1-6	6.879813333	8.038496667	0.044646411	1.158683333
RPPH1	9.826066667	11.45211667	0.016784158	1.62605
RPS19	10.15266333	11.17656333	0.040878628	1.0239
RPS27L	6.73776	7.985196667	0.00650706	1.247436667
SCARNA1	6.18569	7.406243333	0.049331557	1.220553333
SCARNA10	8.75002	10.35652333	0.043127068	1.606503333
SCARNA12	9.590036667	10.92709667	0.000505596	1.33706
SCARNA5	9.326046667	10.70966	0.01083137	1.383613333
SCARNA6	9.29158	10.93077	0.001743211	1.63919
SCARNA7	10.55709333	11.56512333	0.006372725	1.00803
SCARNA8	7.66158	9.21977	0.045956343	1.55819
SCG5	4.872883333	6.653226667	0.016689777	1.780343333
SCIN	4.840283333	6.955756667	0.043960416	2.115473333
SEMA5A	8.406553333	9.571873333	0.010139933	1.16532
03-Sep	4.870143333	6.9245	0.004798961	2.054356667
SLC7A3	5.611423333	8.54911	0.047217463	2.937686667
SNORA14B	8.64935	10.18041	0.023800792	1.53106
SNORA16A	10.76801333	12.34567	0.021956024	1.577656667
SNORA20	11.00365333	12.16392333	0.044373449	1.16027
SNORA23	11.78151	13.17872	0.015427725	1.39721
SNORA24	10.63578	11.67957333	0.006587809	1.043793333
SNORA36A	5.42991	7.159796667	0.038619946	1.729886667
SNORA37	9.070866667	10.49658	0.004811809	1.425713333
SNORA3B	7.512536667	8.583936667	0.010228968	1.0714
SNORA50A	10.25553667	11.25639667	0.043087265	1.00086
SNORA52	7.922526667	9.376156667	0.004225501	1.45363
SNORA54	9.07371	10.26348667	0.006327522	1.189776667
SNORA55	7.51368	8.606846667	0.014652366	1.093166667
SNORA57	9.845093333	11.72496	0.028632107	1.879866667
NORA59B .147(	7.89878	9.113363333	0.046972229	1.214583333
NORA59B .1911	7.89878	9.113363333	0.046972229	1.214583333
SNORA5A	7.19035	8.562423333	0.006308433	1.372073333
SNORA6	7.12769	8.243886667	0.03873327	1.116196667
SNORA60	7.81028	9.98289	0.023639336	2.17261
SNORA62	8.476063333	11.12652667	0.013377969	2.650463333
SNORA65	9.840816667	11.61375333	0.008429764	1.772936667
SNORA67	8.40564	9.55587	0.009879421	1.15023
SNORA68	10.66850333	11.84433667	0.004352053	1.175833333
SNORA71A	8.078836667	9.826036667	0.005608635	1.7472
SNORA71B	8.09232	10.75246333	0.006506272	2.660143333
SNORA71D	7.92425	9.745286667	0.016865099	1.821036667
SNORA73A	11.30723333	12.53301	0.011299142	1.225776667
SNORA8	9.45697	10.48335667	0.000610263	1.026386667
SNORA80E	8.282556667	9.719726667	0.033900257	1.43717
SNORA9	7.773303333	9.465563333	0.02668644	1.69226
SNORD113-4	7.147606667	8.365526667	0.007308571	1.21792
SNORD114-26	6.063526667	7.647946667	0.015387398	1.58442
SNORD114-3	9.821783333	11.6766	0.009628139	1.854816667
SNORD116-1	8.31966	9.390866667	0.033759197	1.071206667
SNORD116-23	6.702643333	7.73212	0.010237396	1.029476667
SNORD116-25	6.137396667	7.139876667	0.037214055	1.00248

SNORD116-29	7.05273	8.597503333	0.010158052	1.544773333
?ORD116-3 .186	10.06163333	11.10885	0.037101082	1.047216667
?ORD116-3 .266	10.06163333	11.10885	0.037101082	1.047216667
SNORD116-4	7.695433333	9.38714	0.014306524	1.691706667
SNORD117	7.820043333	8.900966667	0.008078451	1.080923333
SNORD15A	8.33867	9.42939	0.024779535	1.09072
SNORD1C	10.88253	11.96088333	0.010046699	1.078353333
SNORD25	6.39394	7.808403333	0.033484334	1.414463333
SNORD27	10.01249	11.01879333	0.012212548	1.006303333
SNORD37	6.097343333	7.587346667	0.020845497	1.490003333
SNORD45B	9.62426	10.84894333	0.011495875	1.224683333
SNORD48	9.360723333	10.76543	0.012557316	1.404706667
SNORD54	8.278093333	9.70797	0.003516746	1.429876667
SNORD59B	7.311333333	8.42612	0.031399364	1.114786667
SNORD81	9.974816667	11.42231667	0.002127927	1.4475
SYT11	8.046493333	9.306626667	0.005081316	1.260133333
TDO2	3.98417	8.293736667	0.029840294	4.309566667
TENM1	6.531933333	8.124283333	0.023554707	1.59235
TFAP2C	5.2135	7.49248	0.042494244	2.27898
TIMM8B	6.701846667	7.717073333	0.009145723	1.015226667
TMEM256	7.20938	8.288306667	0.029819853	1.078926667
TP53INP1	8.22494	9.446426667	0.012461348	1.221486667
TRPC6	7.021733333	8.167506667	0.026613721	1.145773333
TYMS	6.003066667	8.718653333	0.012669342	2.715586667
TYROBP	8.737553333	10.10704	0.038344344	1.369486667
UNC5D	6.150053333	8.567016667	0.016653572	2.416963333
UQCRQ	6.775763333	7.79035	0.00586206	1.014586667
VCAN	10.11187	11.90518	0.04451018	1.79331
ZMAT3	8.702983333	10.13674333	0.04012292	1.43376
ZNF561	8.822493333	10.07133333	0.027639258	1.24884
ZNF711	7.785976667	8.963276667	0.027388711	1.1773
ZNRD1	5.991786667	7.028516667	0.035227839	1.03673
ZWINT	5.901303333	6.909453333	0.02981704	1.00815
