# Supplementary material for: Different DNA methylome, transcriptome and histological features in uterine fibroids with and without MED12 mutations
Source: Sci Rep. 2022 May 26;12:8912. doi: 10.1038/s41598-022-12899-7 (PMC9135739; doi:10.1038/s41598-022-12899-7)
Supplement: Supplementary file 4 — Supplementary Table S2. [file 41598_2022_12899_MOESM4_ESM.pdf]

Different DNA methylome, transcriptome and histological features in uterine fibroids with and without MED12 mutations

Ryo Maekawa*, Department of Obstetrics and Gynecology, Yamaguchi University Graduate School of Medicine, Ube, 755-8505 Japan

Shun Sato, Department of Obstetrics and Gynecology, Yamaguchi University Graduate School of Medicine, Ube, 755-8505 Japan

Tetsuro Tamehisa, Department of Obstetrics and Gynecology, Yamaguchi University Graduate School of Medicine, Ube, 755-8505 Japan

Takahiro Sakai, Department of Obstetrics and Gynecology, Yamaguchi University Graduate School of Medicine, Ube, 755-8505 Japan

Takuya Kajimura, Department of Obstetrics and Gynecology, Yamaguchi University Graduate School of Medicine, Ube, 755-8505 Japan

Kotaro Sueoka, Department of Obstetrics and Gynecology, Yamaguchi University Graduate School of Medicine, Ube, 755-8505 Japan

Norihiro Sugino, Department of Obstetrics and Gynecology, Yamaguchi University Graduate School of Medicine, Ube, 755-8505 Japan

Supplemental Table S2. Decreased 233 genes in the MED12m-positive uterine fibroids compared to the myometrium.

Gene symbol	Myometrium (mean log2 value)	MED12m-positive (mean log2 value)	pvalue	fold change (log2)
AASS	9.748826667	8.513436667	0.0029348	-1.23539
ABCA10	7.340423333	4.49177	0.018080599	-2.848653333
ABCA5	7.754806667	6.020443333	0.005725419	-1.734363333
ABCA6	8.034093333	4.80032	0.001323645	-3.233773333
ABCA8	8.1599	4.327913333	0.006306953	-3.831986667
ABCA9	8.429233333	4.89665	0.001115623	-3.532583333
ABCB1	8.197033333	6.82289	0.024063116	-1.374143333
ABCC9	10.50543	9.120323333	0.011091022	-1.385106667
ABI3BP	10.79399333	9.272026667	0.024557259	-1.521966667
ACACB	7.90981	6.905303333	0.00370169	-1.004506667
ACSL4	9.06459	8.06353	0.006570562	-1.00106
ACSS3	9.326046667	5.248833333	0.000388311	-4.077213333
ADAMTSL3	7.57202	5.600943333	0.023311931	-1.971076667
ADH1B	9.478346667	4.666276667	0.012743992	-4.81207
ADIRF	8.698146667	7.54842	0.041175099	-1.149726667
AFF3	8.78357	7.173906667	0.013226125	-1.609663333
AHSA2	8.336113333	7.33248	0.004873482	-1.003633333
ALDH1A1	10.19096667	6.913166667	0.001986791	-3.2778
AMOTL2	8.40608	6.607006667	0.004693265	-1.799073333
ANG	7.447553333	6.34192	0.005346135	-1.105633333
ANKS1B	6.87942	5.744333333	0.011821804	-1.135086667
AOC3	10.15453	8.396973333	0.027391144	-1.757556667
AOX1	6.86736	5.031966667	0.003183359	-1.835393333
APOL1	7.881446667	6.63028	0.003014114	-1.251166667
ARAP2	7.295283333	5.885053333	0.002648784	-1.41023
ARHGAP15	7.412243333	6.401236667	0.014712474	-1.011006667
ARHGEF6	9.222966667	7.82366	0.00354375	-1.399306667
ASPA	7.08642	4.858403333	0.01749132	-2.228016667
ATXN7	9.222236667	8.19572	0.005533205	-1.026516667
BHMT2	7.433436667	6.243226667	0.016690952	-1.19021
C14orf28	8.839153333	7.69549	0.045621477	-1.143663333
C1QTNF7	8.238923333	6.137643333	0.037292676	-2.10128
C1R	9.72456	8.3028	0.009334312	-1.42176
C1S	11.20943	9.065	0.007408962	-2.14443
C1orf198	10.10221	9.05811	0.026454002	-1.0441
C4orf19	7.025293333	5.606596667	0.036545965	-1.418696667
C7	10.84609333	8.060433333	0.002093929	-2.78566
C9orf131	7.29756	6.294483333	0.023575213	-1.003076667
CC2D2B	7.18917	5.973463333	0.010238936	-1.215706667
CCDC109B	8.851763333	7.72472	0.00544665	-1.127043333
CCDC80	12.46395667	11.23359	0.025231921	-1.230366667
CCL5	7.593676667	6.554916667	0.001942654	-1.03876
CD200	9.801933333	8.15543	0.034035378	-1.646503333
CD69	7.18033	5.549303333	0.048656282	-1.631026667
CERS6	10.1512	9.043773333	0.034311807	-1.107426667
CFLAR	9.71957	8.32744	0.003257665	-1.39213
CHL1	7.03769	5.425456667	0.01462042	-1.612233333
CLIP4	8.458183333	7.16574	0.01116939	-1.292443333
CLMN	7.8156	6.583936667	0.025719511	-1.231663333
COL21A1	8.048123333	6.782186667	0.001499394	-1.265936667
CPEB2	9.627703333	7.721633333	0.001304485	-1.90607
CPEB3	6.98581	5.917086667	0.034629733	-1.068723333
CPED1	10.13617	8.913413333	0.00926668	-1.222756667
CPNE8	8.14212	6.99273	0.003526472	-1.14939
CRHBP	6.914143333	5.841423333	0.009298081	-1.07272
CRIM1	9.881896667	8.81794	0.025323488	-1.063956667

CYR61	10.80813333	7.710783333	0.00417467	-3.09735
DEPTOR	9.133593333	7.026576667	0.011974787	-2.107016667
DGKH	9.116733333	7.811033333	0.004032872	-1.3057
DNAJB4	9.91632	8.87551	0.015638228	-1.04081
DPT	8.754213333	5.555486667	0.02532955	-3.198726667
DUSP1	12.37307	9.734406667	0.001090811	-2.638663333
EBF1	9.965006667	8.4465	0.000599648	-1.518506667
ECM2	8.681086667	7.0781	0.001914691	-1.602986667
EDNRB	9.055143333	7.36309	0.001399122	-1.692053333
EFEMP1	9.615313333	7.239266667	0.001179125	-2.376046667
EGR1	10.71887667	7.99405	0.033420743	-2.724826667
EPB41L2	9.636013333	8.315026667	0.018556499	-1.320986667
FAM46A	8.445166667	7.170696667	0.000906329	-1.27447
FAM69A	9.19713	8.045856667	0.008738778	-1.151273333
FAXDC2	9.735333333	8.51072	0.032298399	-1.224613333
FGL2	9.41001	7.734396667	0.001567396	-1.675613333
FHL5	9.108466667	6.731316667	0.006663944	-2.37715
FLJ42393	8.218886667	6.40452	0.001819833	-1.814366667
FRZB	9.703836667	8.005413333	0.005854771	-1.698423333
GAB1	10.19798667	8.794046667	0.015286282	-1.40394
GALNT7	8.36433	7.28522	0.042440048	-1.07911
GLS	9.908836667	8.854713333	0.01970696	-1.054123333
GNAI1	8.6754	7.621526667	0.000622655	-1.053873333
GPC4	7.983066667	6.3656	0.015330124	-1.617466667
GPM6A	7.34808	5.783146667	0.001344819	-1.564933333
GREB1L	6.69534	4.655383333	0.043117835	-2.039956667
GSAP	6.948843333	5.758056667	0.035665114	-1.190786667
GSTM5	8.850226667	4.947366667	0.014110955	-3.90286
GUCY1A3	9.418113333	8.24803	0.029401323	-1.170083333
GUCY1B3	10.16829333	8.751096667	0.005349633	-1.417196667
GZMK	6.722366667	4.967633333	0.046086194	-1.754733333
HBEGF	8.74029	6.431773333	0.008062826	-2.308516667
HERC3	8.821906667	7.64152	0.008820107	-1.180386667
HEYL	8.528646667	7.242666667	0.041636057	-1.28598
HLF	8.24091	6.698243333	0.005319345	-1.542666667
IFI44L	8.900826667	7.69114	0.038514901	-1.209686667
IL6ST	11.07552	10.04637333	0.012924247	-1.029146667
INADL	8.067313333	6.819323333	0.018342488	-1.24799
INPP4B	8.710003333	7.215613333	0.031661732	-1.49439
INSIG2	8.89284	7.840563333	0.032186703	-1.052276667
ITGA6	10.42291333	9.31295	0.020967283	-1.109963333
JAZF1	9.833296667	8.408583333	0.036377936	-1.424713333
JPH1	8.617016667	6.860613333	0.008477701	-1.756403333
JUN	10.42587333	8.730743333	0.009021332	-1.69513
KIAA0040	7.199153333	6.07056	0.03856741	-1.128593333
KLF4	8.432523333	6.46581	0.004513677	-1.966713333
KLF8	7.466816667	6.411433333	0.034989308	-1.055383333
KRT19	7.85503	6.42584	0.038260875	-1.42919
LATS2	9.084853333	8.070503333	0.012296787	-1.01435
LDB2	10.66204333	9.415726667	0.004903013	-1.246316667
LGR4	9.009346667	7.91016	0.006760149	-1.099186667
LIFR	11.14448667	8.732326667	0.000491178	-2.41216
LINC00472	6.960333333	5.778956667	0.027812049	-1.181376667
LINC00969	7.286803333	6.152106667	0.005162821	-1.134696667
LINC01140	7.00869	5.876406667	0.012523059	-1.132283333
LOC100131541	6.88243	5.44609	0.027143026	-1.43634
LOC100287934	7.892086667	6.668026667	0.011422015	-1.22406
LPCAT2	9.70569	8.485106667	0.000966114	-1.220583333

LRRFIP1	11.15722	9.903573333	0.026669617	-1.253646667
LRRFIP2	8.715023333	7.633303333	0.014775272	-1.08172
LY6G5B	8.959383333	7.665793333	0.018470132	-1.29359
LYST	9.200453333	7.995356667	0.002998859	-1.205096667
MAMDC2	10.55758	9.55304	0.008006751	-1.00454
MAML2	8.605423333	7.432356667	0.012255393	-1.173066667
MAOA	8.27904	6.978996667	0.013337174	-1.300043333
MAPK10	10.21522667	8.310226667	0.035793222	-1.905
02-Mar	7.749756667	6.589513333	0.001706167	-1.160243333
MBNL2	11.64991333	10.52447667	0.009375071	-1.125436667
MBP	7.475663333	6.313596667	0.000614502	-1.162066667
MEOX2	7.841953333	6.568373333	0.017026569	-1.27358
MGARP	6.88712	5.68053	0.00606101	-1.20659
MIR186	6.65969	5.19376	0.041972066	-1.46593
MIR99AHG	9.608566667	8.012783333	0.025351503	-1.595783333
MLLT4	8.64622	7.410166667	0.014948245	-1.236053333
MTUS1	8.254003333	7.031096667	0.011582925	-1.222906667
MYCT1	8.92133	7.677266667	0.003054672	-1.244063333
MYOM1	7.71292	5.911676667	0.010191801	-1.801243333
N4BP2L1	6.657516667	5.47363	0.01572497	-1.183886667
NAALADL2	7.878583333	6.725446667	0.008655271	-1.153136667
NAV3	7.42607	5.976093333	0.04890671	-1.449976667
NDRG1	10.24195667	8.96798	0.00714335	-1.273976667
NDRG2	9.017893333	8.007146667	0.015228984	-1.010746667
NEGR1	8.516783333	6.123956667	0.023808376	-2.392826667
NFIB	11.30307	10.11457667	0.043950432	-1.188493333
NLGN1	6.89103	5.237363333	0.043088299	-1.653666667
NR3C1	9.118153333	8.081223333	0.034791761	-1.03693
NR3C2	7.721546667	6.32238	0.006993641	-1.399166667
NR4A1	9.57817	7.145743333	0.025790666	-2.432426667
NR4A2	6.9441	5.623016667	0.014849262	-1.321083333
NTN4	8.914676667	7.668123333	0.00186857	-1.246553333
OLFML1	9.096043333	6.291046667	0.002588982	-2.804996667
OMD	9.024366667	6.802826667	0.007321983	-2.22154
OR2A4	7.690326667	6.496646667	0.046024377	-1.19368
PDE10A	8.298633333	6.807646667	0.000235971	-1.490986667
PDE2A	7.43672	6.35326	0.012671798	-1.08346
PHACTR2	9.301916667	8.245	0.004534503	-1.056916667
PHF11	7.6567	6.596633333	0.040091296	-1.060066667
PIWIL4	6.726026667	5.71492	0.021196293	-1.011106667
PLA2G4A	7.7935	5.952393333	0.01393893	-1.841106667
PLBD1	8.25081	7.215186667	0.047702353	-1.035623333
PLCB4	8.51734	6.79016	0.000483418	-1.72718
PLCE1	9.55058	7.642446667	0.009579465	-1.908133333
PLEKHH2	10.03808667	8.117016667	0.005030583	-1.92107
PODN	9.105926667	7.596653333	0.047533908	-1.509273333
PPARG	7.52326	6.400166667	0.019026832	-1.123093333
PPM1K	9.096873333	7.502313333	0.035725793	-1.59456
PPP1R15A	8.247773333	7.111893333	0.024170764	-1.13588
PRICKLE1	9.418966667	7.998136667	0.002842177	-1.42083
PRKAA2	7.94503	6.854306667	0.041386737	-1.090723333
PROS1	9.169886667	8.14633	0.024283222	-1.023556667
PROS2P	8.810276667	7.688933333	0.026776517	-1.121343333
PTER	8.196223333	7.13398	0.000344475	-1.062243333
PTGR1	8.62082	7.619456667	0.028579938	-1.001363333
PYGM	7.5531	6.320143333	0.043452611	-1.232956667
RAPGEF4	8.307466667	7.065683333	0.001259493	-1.241783333
RASA4	8.612376667	7.324823333	0.037281812	-1.287553333

RASSF9	7.86323	6.511076667	0.022552194	-1.352153333
RCAN2	8.935136667	7.642163333	0.000331855	-1.292973333
REPS2	9.059516667	7.45361	0.009336218	-1.605906667
RFTN2	8.464223333	6.733506667	0.011509489	-1.730716667
RHOBTB3	8.975396667	7.740023333	0.00564689	-1.235373333
RNF180	8.979106667	7.45919	0.001829906	-1.519916667
RORA	8.60054	6.942406667	0.003203566	-1.658133333
RSPO1	9.43872	7.623133333	0.046087151	-1.815586667
SAMD4A	8.821523333	7.751276667	0.01851205	-1.070246667
SCML1	7.331853333	6.067396667	0.037008665	-1.264456667
SCN7A	9.34427	5.178493333	0.000385586	-4.165776667
SDC4	9.481313333	8.312136667	0.002357617	-1.169176667
SELE	7.581963333	5.639383333	0.037248865	-1.94258
SELP	8.126846667	6.969216667	0.022381909	-1.15763
SERPINB9	7.912003333	6.750516667	0.006547426	-1.161486667
SERPING1	11.22260333	9.969053333	0.017911776	-1.25355
SLC16A4	6.84775	5.658933333	0.00823011	-1.188816667
SLC16A9	7.886943333	6.366746667	0.033658058	-1.520196667
SLC25A27	8.232906667	6.717246667	0.013954891	-1.51566
SLC2A3	9.607473333	8.451123333	0.010199765	-1.15635
SLC37A3	10.05817333	8.90076	0.003238978	-1.157413333
SLC6A16	6.773103333	5.71736	0.006996246	-1.055743333
SLC7A2	8.707266667	6.995096667	0.009578302	-1.71217
SLFN11	8.294676667	6.530973333	0.00022178	-1.763703333
SLFN12L	6.9645	5.888536667	0.014232265	-1.075963333
SND1-IT1	7.07089	5.84881	0.010108399	-1.22208
SNRPA1	7.281813333	5.906386667	0.000857538	-1.375426667
SNX25	8.047483333	7.03118	0.001540428	-1.016303333
SORBS2	7.92815	6.649746667	0.00467921	-1.278403333
SRPX	9.371483333	7.61439	0.038304007	-1.757093333
STEAP2	8.041236667	6.12969	5.87E-05	-1.911546667
STEAP4	9.346616667	6.501563333	0.010813621	-2.845053333
STXBP5	8.5045	7.296296667	0.003360091	-1.208203333
SYDE2	7.991116667	6.794253333	0.013150244	-1.196863333
SYPL1	10.17364667	9.15191	0.006087493	-1.021736667
SYTL4	9.653346667	7.96264	0.007053099	-1.690706667
TEX15	9.14342	6.65959	0.009019805	-2.48383
TFPI	8.6311	7.02616	0.013800323	-1.60494
TGFBR2	10.80762333	9.75096	0.015684196	-1.056663333
THBD	8.354823333	7.033913333	0.002774056	-1.32091
TJP1	11.67742	10.61753333	0.038239876	-1.059886667
TLR3	8.68699	6.911113333	0.017516552	-1.775876667
TMEM144	7.021736667	5.76735	0.03616886	-1.254386667
TMTC1	8.363153333	7.339756667	0.02720728	-1.023396667
TNXB	9.174756667	7.453253333	0.00964538	-1.721503333
TRIB1	8.281366667	6.951846667	0.007548831	-1.32952
TRPC4	8.41728	6.195093333	0.008327742	-2.222186667
TSPAN7	10.44686	9.068643333	0.010205712	-1.378216667
TTC39C	8.012956667	6.89252	0.005036023	-1.120436667
TTLL7	8.6943	7.626946667	0.030152677	-1.067353333
TXNRD1	9.73557	8.67603	0.012465588	-1.05954
ULK4	7.197413333	5.921273333	0.014888962	-1.27614
UST	7.880436667	6.874803333	0.006556146	-1.005633333
WEE1	9.66709	7.38419	0.000495552	-2.2829
WWC2	8.69584	7.576576667	0.040384387	-1.119263333
ZBTB20	9.87607	8.79829	0.008862945	-1.07778
ZC3H6	9.231126667	8.20179	0.038952625	-1.029336667
ZFHX4	8.06592	6.079796667	0.028101837	-1.986123333

ZFPM2	9.931136667	7.726116667	0.035697956	-2.20502
ZNF204P	6.952393333	4.382143333	0.038119671	-2.57025
ZNF483	7.83777	6.56585	0.008555543	-1.27192
