# Supplementary material for: Different DNA methylome, transcriptome and histological features in uterine fibroids with and without MED12 mutations
Source: Sci Rep. 2022 May 26;12:8912. doi: 10.1038/s41598-022-12899-7 (PMC9135739; doi:10.1038/s41598-022-12899-7)
Supplement: Supplementary file 5 — Supplementary Table S3. [file 41598_2022_12899_MOESM5_ESM.pdf]

Different DNA methylome, transcriptome and histological features in uterine fibroids with and without MED12 mutations

Ryo Maekawa*, Department of Obstetrics and Gynecology, Yamaguchi University Graduate School of Medicine, Ube, 755-8505 Japan

Shun Sato, Department of Obstetrics and Gynecology, Yamaguchi University Graduate School of Medicine, Ube, 755-8505 Japan

Tetsuro Tamehisa, Department of Obstetrics and Gynecology, Yamaguchi University Graduate School of Medicine, Ube, 755-8505 Japan

Takahiro Sakai, Department of Obstetrics and Gynecology, Yamaguchi University Graduate School of Medicine, Ube, 755-8505 Japan

Takuya Kajimura, Department of Obstetrics and Gynecology, Yamaguchi University Graduate School of Medicine, Ube, 755-8505 Japan

Kotaro Sueoka, Department of Obstetrics and Gynecology, Yamaguchi University Graduate School of Medicine, Ube, 755-8505 Japan

Norihiro Sugino, Department of Obstetrics and Gynecology, Yamaguchi University Graduate School of Medicine, Ube, 755-8505 Japan

Supplemental Table S3. Increased 110 genes in the MED12m-negative uterine fibroids compared to the myometrium.

Gene symbol	Myometrium (mean log2 value)	MED12m-negative (mean log2 value)	pvalue	fold change (log2)
ADAM12	5.422913333	7.4077	0.000701842	1.984786667
ASB5	4.924286667	7.799378889	0.000943875	2.875092222
ATP8B4	7.517466667	9.132755556	0.004462608	1.615288889
BCL11A	5.50903	7.279942222	0.005534676	1.770912222
CAPN6	8.36719	11.83462889	0.016983595	3.467438889
CCND1	8.468586667	10.04997778	0.020288854	1.581391111
CDH8	5.59772	7.049057778	0.010478488	1.451337778
CHI3L1	5.329746667	6.75988	0.039259895	1.430133333
CHRM2	5.075763333	6.746512222	0.015996281	1.670748889
CKS2	5.67796	8.119026667	0.000614882	2.441066667
CLVS2	5.999313333	8.098232222	0.022211317	2.098918889
CPNE5	5.858046667	7.3004	0.003552735	1.442353333
CRISPLD1	6.514976667	7.531031111	0.008144123	1.016054444
DCX	5.438426667	8.669886667	0.000631261	3.23146
DKK2	6.004376667	7.510278889	0.004274208	1.505902222
DNAH14	5.40168	7.84864	0.00015466	2.44696
EDA2R	7.431523333	8.765076667	0.013544941	1.333553333
EFNA5	5.733176667	6.777223333	0.008392383	1.044046667
EYA4	5.44283	7.907221111	0.001653914	2.464391111
FAM171A2	6.032046667	7.220955556	0.001501585	1.188908889
FAM229B	7.626286667	8.786267778	0.002406961	1.159981111
FKBP2	8.1321	9.14771	0.000480323	1.01561
GALNT13	5.601096667	8.309974444	0.000623235	2.708877778
GPR34	6.000876667	7.163473333	0.00225998	1.162596667
GRIN2A	5.58568	7.030981111	0.000518449	1.445301111
H19	9.388316667	11.20927222	0.013347768	1.820955556
HIST1H1D	7.30653	8.601001111	0.012609109	1.294471111
HIST1H2BH	6.769416667	7.861736667	0.028463551	1.09232
HIST1H2BM	5.722036667	6.879416667	0.000888068	1.15738
HIST1H3B	5.677053333	6.945531111	0.003726073	1.268477778
HIST1H3F	5.969266667	7.046711111	0.000429715	1.077444444
HIST1H4C	8.927526667	10.79441444	0.000325308	1.866887778
HIST1H4F	7.090936667	8.24444	0.010191761	1.153503333
HIST1H4J	8.189606667	9.946696667	4.02E-06	1.75709
HIST1H4K	8.207853333	10.19739778	2.38E-06	1.989544444
HMGA2	5.49236	6.716613333	0.011589974	1.224253333
HOXA13	7.005936667	8.123315556	9.67E-05	1.117378889
HSD17B6	8.23956	9.890976667	0.042435761	1.651416667
IGSF3	5.902806667	7.144214444	0.002306033	1.241407778
KIF5C	6.070986667	7.359553333	0.007779155	1.288566667
LEFTY2	7.85181	9.508187778	0.022891948	1.656377778
LOC101060256	5.633333333	6.728634444	0.000157943	1.095301111
LPAR4	6.359553333	7.835114444	0.007025299	1.475561111
LRRC69	4.68204	7.157974444	0.011863159	2.475934444
LRRC7	4.799883333	7.414542222	0.005407631	2.614658889
LRRTM1	5.33428	6.945601111	0.003223109	1.611321111
MFAP2	7.904486667	8.978418889	0.004164922	1.073932222
MMP11	6.63188	7.906231111	0.008585485	1.274351111
MMP16	6.732336667	8.255667778	0.007342644	1.523331111
MSL3P1	6.340436667	7.404744444	0.043781406	1.064307778
NCAM2	5.909763333	7.072071111	0.012123662	1.162307778
NDNF	6.397543333	7.439558889	0.047564835	1.042015556
NETO2	6.020346667	7.612875556	0.000343125	1.592528889
NKAIN4	5.65879	6.801412222	0.033816714	1.142622222
OSR1	5.74873	7.133701111	0.005040373	1.384971111
PAK3	6.940193333	8.27935	0.004992667	1.339156667
PAPPA2	5.771596667	7.323244444	0.025240852	1.551647778
PCDH10	5.84376	7.062173333	0.000653765	1.218413333
PCDH20	7.04882	8.106173333	0.030432804	1.057353333
PKP2	7.0192	8.239058889	0.000640676	1.219858889
PLAG1	5.10123	7.559017778	0.002398997	2.457787778

PMAIP1	4.94694	6.870595556	0.003350563	1.923655556
PPP1R1A	6.811956667	8.019911111	0.010324418	1.207954444
PRR16	6.775833333	8.25879	0.004503623	1.482956667
PTCHD4	7.070283333	8.72105	0.001339594	1.650766667
RNU11	8.97137	10.25805333	0.00166812	1.286683333
RPPH1	9.826066667	11.13882778	0.01717141	1.312761111
SATB2	7.33533	9.974545556	4.43E-05	2.639215556
SCARNA11	7.65175	8.697533333	0.021932504	1.045783333
SCARNA12	9.590036667	10.63218	0.000108799	1.042143333
SCARNA6	9.29158	10.39910444	0.001932627	1.107524444
SFRP2	5.591186667	7.057392222	0.039173286	1.466205556
SLC39A8	7.775756667	9.155683333	0.017471352	1.379926667
SLC7A3	5.611423333	7.924423333	0.012477223	2.313
SNORA23	11.78151	13.21359778	0.004152198	1.432087778
SNORA2A	6.04987	8.897023333	1.77E-05	2.847153333
SNORA33	6.533803333	7.601906667	0.015856469	1.068103333
SNORA37	9.070866667	10.13583111	0.000903604	1.064964444
SNORA38B	8.027563333	10.50262778	0.002303251	2.475064444
SNORA57	9.845093333	11.35370778	9.44E-05	1.508614444
SNORA59B .14700	7.89878	8.985842222	0.011437896	1.087062222
SNORA59B .19117	7.89878	8.985842222	0.011437896	1.087062222
SNORA60	7.81028	9.468008889	0.019911221	1.657728889
SNORA62	8.476063333	10.17407667	0.004339143	1.698013333
SNORA65	9.840816667	11.18672778	0.005244587	1.345911111
SNORA71A	8.078836667	9.292916667	0.002795619	1.21408
SNORA71B	8.09232	11.08355333	0.000969782	2.991233333
SNORA71D	7.92425	9.480146667	0.003575136	1.555896667
SNORA80E	8.282556667	9.289277778	0.033102848	1.006721111
SNORD116-23	6.702643333	7.878206667	0.006025672	1.175563333
SNORD116-25	6.137396667	7.326318889	0.038669135	1.188922222
SNORD116-26	6.434883333	7.862491111	0.014811745	1.427607778
SNORD116-29	7.05273	8.502983333	0.006974389	1.450253333
SNORD116-4	7.695433333	9.295413333	0.006483356	1.59998
SNORD25	6.39394	7.485636667	0.007183026	1.091696667
SNORD27	10.01249	11.12710889	0.001640929	1.114618889
SNORD42A	9.614533333	10.82339333	0.001341457	1.20886
SNORD45B	9.62426	10.68610556	0.011416906	1.061845556
SNORD48	9.360723333	10.65342667	0.010994434	1.292703333
SNORD52	7.81795	9.055483333	0.000381233	1.237533333
SNORD81	9.974816667	11.02102222	0.001520869	1.046205556
TERC	6.78311	7.952556667	5.69E-05	1.169446667
TFAP2C	5.2135	6.669372222	0.042703281	1.455872222
TGFBI	8.991106667	10.39899222	0.003983774	1.407885556
TOP2A	5.22348	6.724165556	0.009872035	1.500685556
TYMS	6.003066667	7.551388889	0.034059866	1.548322222
TYROBP	8.737553333	9.78986	0.00747327	1.052306667
WIF1	5.266313333	7.615372222	0.007962095	2.349058889
XG	6.00675	7.939318889	0.006179436	1.932568889
ZNF663P	5.336316667	7.03356	0.003363565	1.697243333
