# Supplementary material for: Different DNA methylome, transcriptome and histological features in uterine fibroids with and without MED12 mutations
Source: Sci Rep. 2022 May 26;12:8912. doi: 10.1038/s41598-022-12899-7 (PMC9135739; doi:10.1038/s41598-022-12899-7)
Supplement: Supplementary file 6 — Supplementary Table S4. [file 41598_2022_12899_MOESM6_ESM.pdf]

Different DNA methylome, transcriptome and histological features in uterine fibroids with and without MED12 mutations

Ryo Maekawa*, Department of Obstetrics and Gynecology, Yamaguchi University Graduate School of Medicine, Ube, 755-8505 Japan

Shun Sato, Department of Obstetrics and Gynecology, Yamaguchi University Graduate School of Medicine, Ube, 755-8505 Japan

Tetsuro Tamehisa, Department of Obstetrics and Gynecology, Yamaguchi University Graduate School of Medicine, Ube, 755-8505 Japan

Takahiro Sakai, Department of Obstetrics and Gynecology, Yamaguchi University Graduate School of Medicine, Ube, 755-8505 Japan

Takuya Kajimura, Department of Obstetrics and Gynecology, Yamaguchi University Graduate School of Medicine, Ube, 755-8505 Japan

Kotaro Sueoka, Department of Obstetrics and Gynecology, Yamaguchi University Graduate School of Medicine, Ube, 755-8505 Japan

Norihiro Sugino, Department of Obstetrics and Gynecology, Yamaguchi University Graduate School of Medicine, Ube, 755-8505 Japan

Supplemental Table S4. Decreased 207 genes in the MED12m-negative uterine fibroids compared to the myometrium.

Gene symbol	Myometrium (mean log2 value)	MED12m-negative (mean log2 value)	pvalue	fold change (log2)
AASS	9.748826667	8.498497778	0.000132049	-1.250328889
ABCA10	7.340423333	4.554222222	0.026809795	-2.786201111
ABCA5	7.754806667	6.057642222	7.67E-06	-1.697164444
ABCA6	8.034093333	5.746515556	0.000116427	-2.287577778
ABCA8	8.1599	6.024598889	0.022305602	-2.135301111
ABCA9	8.429233333	5.852578889	0.000651942	-2.576654444
ABCB1	8.197033333	7.189256667	0.017632403	-1.007776667
ABCC4	7.76726	6.652903333	0.000493735	-1.114356667
ABLIM1	9.03055	7.562604444	2.87E-05	-1.467945556
ACSL1	8.69993	7.695111111	0.02589715	-1.004818889
ACSS3	9.326046667	8.035497778	0.004766734	-1.290548889
ADGRD1	7.95295	6.68189	0.002061602	-1.27106
ADH1B	9.478346667	5.788143333	0.029677593	-3.690203333
AFAP1L2	7.98293	6.910837778	0.007867628	-1.072092222
AFF3	8.78357	7.411196667	0.005220642	-1.372373333
AGTR2	8.748006667	5.801621111	9.71E-05	-2.946385556
AKAP6	7.994206667	6.602044444	0.000613454	-1.392162222
ALDH1A1	10.19096667	6.984896667	0.002783445	-3.20607
ALDH1A2	8.496923333	7.278153333	0.008081932	-1.21877
ALDH1B1	11.06810333	9.626967778	0.017594696	-1.441135556
AMIGO2	9.199903333	8.109792222	0.031283752	-1.090111111
AMOTL2	8.40608	6.875642222	0.002987694	-1.530437778
AOX1	6.86736	5.043213333	0.000906441	-1.824146667
APOD	11.42763	10.05801556	0.006326432	-1.369614444
ARAP2	7.295283333	5.973468889	1.53E-06	-1.321814444
ATP8B1	9.451416667	8.346408889	0.004000788	-1.105007778
BAMBI	8.82291	7.706688889	0.043098458	-1.116221111
C1R	9.72456	8.618787778	0.006012139	-1.105772222
C1S	11.20943	9.484965556	0.000772061	-1.724464444
C1orf198	10.10221	8.684046667	0.014749007	-1.418163333
C3	8.41489	6.520203333	0.000358241	-1.894686667
C7	10.84609333	7.565051111	0.001043128	-3.281042222
CCDC3	8.90116	7.52184	0.000329638	-1.37932
CCDC80	12.46395667	11.42876222	0.003140985	-1.035194444
CD44	12.349	11.33142222	0.033581124	-1.017577778
CITED2	10.00416	8.843867778	0.001682688	-1.160292222
CLSTN2	8.738923333	7.26077	0.032134486	-1.478153333
CMKLR1	8.30859	7.219317778	0.022617854	-1.089272222
CNTN1	8.566746667	6.50303	1.51E-05	-2.063716667
COL14A1	10.77778333	9.529687778	0.001097547	-1.248095556
CPEB2	9.627703333	7.908711111	0.000584052	-1.718992222
CPED1	10.13617	8.253556667	0.000785487	-1.882613333
CPXM2	7.462683333	6.228508889	0.00314116	-1.234174444
CTGF	10.10648	8.488077778	0.000898827	-1.618402222
CTTNBP2	8.739303333	7.429447778	0.022500159	-1.309855556
CYR61	10.80813333	8.732036667	1.40E-05	-2.076096667
DAPK1	8.527336667	7.447178889	0.023323536	-1.080157778
DCN	12.50683667	11.22622333	0.001512539	-1.280613333
DDAH1	9.504063333	8.49092	0.013341036	-1.013143333
DFNA5	7.320933333	6.28251	0.004183233	-1.038423333
DGKH	9.116733333	7.818597778	0.001567056	-1.298135556
DNAJB5	10.23268667	9.029805556	0.026462018	-1.202881111
DOCK5	7.496656667	6.366467778	1.12E-05	-1.130188889
DPT	8.754213333	6.553092222	7.55E-05	-2.201121111
DUSP1	12.37307	10.67743333	0.000342419	-1.695636667
EBF1	9.965006667	8.278587778	7.90E-06	-1.686418889
ECM2	8.681086667	7.46916	0.002202375	-1.211926667
EDIL3	10.46289333	9.00105	0.008257024	-1.461843333
EDNRB	9.055143333	7.84054	0.00241874	-1.214603333

EFEMP1	9.615313333	7.132602222	1.47E-05	-2.482711111
EGFR	8.461296667	7.055672222	0.001037095	-1.405624444
ELOVL7	7.551056667	6.402684444	0.003099401	-1.148372222
ENPEP	10.34443333	9.131013333	0.012558354	-1.21342
EPB41L2	9.636013333	8.425304444	1.96E-06	-1.210708889
EPHA3	8.78201	7.493571111	0.007742066	-1.288438889
FAM160A1	6.875613333	5.668081111	0.026578541	-1.207532222
FAM189A2	7.89901	6.189863333	3.29E-05	-1.709146667
FBLN1	9.9259	8.752152222	0.029371035	-1.173747778
FBLN5	10.95694333	9.855872222	0.005537197	-1.101071111
FGL2	9.41001	8.405415556	0.005569997	-1.004594444
FHL5	9.108466667	7.601053333	0.003184707	-1.507413333
FIGN	7.309243333	5.966118889	1.77E-05	-1.343124444
FLJ42393	8.218886667	6.408244444	0.000250754	-1.810642222
FMO2	7.061023333	5.133446667	0.000196833	-1.927576667
FOXP2	8.660763333	6.68976	0.029728963	-1.971003333
FRZB	9.703836667	8.612416667	0.011852363	-1.09142
GLI3	9.33667	8.102321111	0.003719229	-1.234348889
GPC4	7.983066667	6.674431111	0.006625227	-1.308635556
GPM6A	7.34808	6.078437778	3.39E-05	-1.269642222
GPNMB	11.10768	9.698975556	0.038499314	-1.408704444
GRAMD3	9.16672	8.029064444	0.014297508	-1.137655556
GSTM5	8.850226667	5.658325556	0.029162652	-3.191901111
GUCY1A3	9.418113333	8.331468889	0.02794322	-1.086644444
GZMK	6.722366667	5.264433333	0.000207884	-1.457933333
HBEGF	8.74029	7.164017778	0.019090512	-1.576272222
HERC3	8.821906667	7.808505556	1.32E-05	-1.013401111
HEYL	8.528646667	7.27463	0.00569278	-1.254016667
HIVEP2	8.654776667	7.399735556	0.001128229	-1.255041111
HPSE2	7.376906667	6.125934444	0.009538076	-1.250972222
HTATIP2	6.761046667	5.317862222	0.042086294	-1.443184444
IFITM1	10.68279333	9.622581111	0.016713883	-1.060212222
IGSF10	6.733506667	5.312835556	0.003823403	-1.420671111
INMT	7.809076667	6.713638889	0.002494038	-1.095437778
INPP4B	8.710003333	7.443985556	0.000318069	-1.266017778
ITGA11	10.12378	8.907702222	0.03160652	-1.216077778
ITGA6	10.42291333	9.231872222	0.010255903	-1.191041111
KIF21A	8.036183333	6.897333333	0.013351427	-1.13885
KLF4	8.432523333	6.933992222	0.000779189	-1.498531111
KRT19	7.85503	6.413957778	0.037247762	-1.441072222
LGR4	9.009346667	7.866466667	0.002264441	-1.14288
LIFR	11.14448667	9.591141111	9.23E-05	-1.553345556
LINC00260	7.804546667	6.18547	0.004653515	-1.619076667
LOC100130428	7.141806667	6.139781111	0.009479891	-1.002025556
LOC100130876	9.45444	7.625677778	0.040382196	-1.828762222
LOC100131541	6.88243	5.610717778	0.003527803	-1.271712222
LOC100133299	7.78315	6.631448889	0.000278853	-1.151701111
LOC100287934	7.892086667	6.187191111	0.000110054	-1.704895556
LONRF2	10.88587333	9.874014444	0.011530548	-1.011858889
LPAR1	9.696726667	8.435901111	0.000216991	-1.260825556
LRRC16A	8.0481	6.881626667	0.016254843	-1.166473333
LRRFIP1	11.15722	9.917346667	0.012575029	-1.239873333
LTBP1	10.64839	9.378725556	3.03E-05	-1.269664444
LUM	11.71373	10.56100333	0.039303678	-1.152726667
LY6G5B	8.959383333	7.360584444	0.009997789	-1.598798889
MAP3K8	8.43389	7.176256667	0.046094917	-1.257633333
MEOX2	7.841953333	6.772162222	0.031401754	-1.069791111
MIR186	6.65969	5.266391111	0.000373931	-1.393298889
MIR218-1	7.101053333	5.720876667	0.000171761	-1.380176667
NDRG1	10.24195667	9.211838889	0.010500729	-1.030117778
NEGR1	8.516783333	6.315065556	1.49E-05	-2.201717778

NFIA	10.45742667	9.405158889	0.00143187	-1.052267778
NFIB	11.30307	10.20489444	0.001277817	-1.098175556
NR4A1	9.57817	7.878101111	0.032627059	-1.700068889
NT5DC3	9.51782	8.319572222	0.029518728	-1.198247778
OLFML1	9.096043333	6.76877	3.88E-05	-2.327273333
OMD	9.024366667	6.913476667	0.000455821	-2.11089
OSBPL10	9.229663333	8.095303333	0.008450643	-1.13436
PAPSS2	8.425596667	7.380542222	0.000229115	-1.045054444
PAWR	9.456773333	8.341748889	0.007410991	-1.115024444
PCOLCE	8.863696667	7.57919	0.007675389	-1.284506667
PDE3A	9.004066667	7.902612222	0.001753769	-1.101454444
PDE5A	9.557693333	8.187027778	0.014994714	-1.370665556
PDGFRA	10.59073	9.226125556	0.026211528	-1.364604444
PKD2	11.24644333	10.21355222	4.35E-07	-1.032891111
PLA2R1	9.14131	7.888062222	0.012713897	-1.253247778
PLAT	9.71353	8.587435556	0.002007018	-1.126094444
PLBD1	8.25081	7.053526667	0.000298857	-1.197283333
PLCB4	8.51734	7.05956	0.000206568	-1.45778
PLCE1	9.55058	7.950046667	0.00095946	-1.600533333
PLEKHH2	10.03808667	8.802965556	0.000526076	-1.235121111
PLTP	9.86619	8.790262222	0.002103441	-1.075927778
PODN	9.105926667	8.074173333	0.018887765	-1.031753333
PPM1K	9.096873333	7.825687778	0.047935233	-1.271185556
PPP2R2C	7.871763333	6.637963333	0.003202389	-1.2338
PRELP	10.47603667	9.24461	0.03400747	-1.231426667
PRICKLE1	9.418966667	7.897606667	0.000270236	-1.52136
PRKAA2	7.94503	6.930364444	0.015356246	-1.014665556
PRRX1	10.48800667	9.211337778	0.000252542	-1.276668889
RAPGEF4	8.307466667	7.223794444	2.70E-05	-1.083672222
RASSF2	10.31548667	8.994604444	0.01639374	-1.320882222
RASSF9	7.86323	6.667593333	0.003182623	-1.195636667
RBP1	7.9855	6.736013333	0.014266285	-1.249486667
RCAN2	8.935136667	7.74768	2.47E-05	-1.187456667
RERGL	6.793236667	5.518698889	0.018780144	-1.274537778
RND3	10.30471333	9.278045556	0.005794858	-1.026667778
RNF180	8.979106667	7.604663333	8.61E-05	-1.374443333
RORA	8.60054	7.413663333	2.38E-06	-1.186876667
RSPO1	9.43872	7.67632	0.003530381	-1.7624
RYR3	8.2539	7.229912222	0.026986942	-1.023987778
SCN7A	9.34427	6.258091111	0.000190584	-3.086178889
SELP	8.126846667	6.891762222	7.92E-05	-1.235084444
SEMA3D	7.526783333	6.287042222	0.004720837	-1.239741111
SERPING1	11.22260333	10.11026889	0.004211177	-1.112334444
SFT2D2	9.269486667	8.180681111	0.008894854	-1.088805556
SHTN1	8.097226667	7.086937778	6.93E-07	-1.010288889
SLC16A4	6.84775	5.559501111	0.0002877	-1.288248889
SLC25A27	8.232906667	7.137058889	0.022886825	-1.095847778
SLC37A3	10.05817333	8.743185556	0.000731698	-1.314987778
SLC6A16	6.773103333	5.735912222	0.004347301	-1.037191111
SLC7A2	8.707266667	7.51631	0.022947665	-1.190956667
SLCO2A1	9.587043333	8.107962222	0.002822897	-1.479081111
SLFN11	8.294676667	7.173256667	9.73E-06	-1.12142
SLIT2	10.37347	8.870693333	0.007433838	-1.502776667
SLITRK3	7.09005	5.431002222	0.034404355	-1.659047778
SMOC2	10.53375333	9.102764444	0.021972728	-1.430988889
SNCA	9.140423333	7.893168889	0.048976498	-1.247254444
SND1-IT1	7.07089	5.504001111	0.002106572	-1.566888889
SNRPA1	7.281813333	6.040822222	0.000103014	-1.240991111
SORBS2	7.92815	6.893941111	0.002147877	-1.034208889
SPON1	10.00346667	8.102867778	0.001468981	-1.900598889
SRPX	9.371483333	8.047292222	0.035516285	-1.324191111

STARD9	7.830563333	6.606735556	0.002670367	-1.223827778
STEAP2	8.041236667	6.626337778	1.06E-05	-1.414898889
STEAP4	9.346616667	7.242648889	0.01583579	-2.103967778
SVEP1	9.305393333	7.27238	0.000134905	-2.033013333
SYDE2	7.991116667	6.926221111	0.008730556	-1.064895556
SYTL4	9.653346667	8.054754444	0.003402188	-1.598592222
TACR1	6.70388	5.653101111	0.00099566	-1.050778889
TAS2R20	6.773876667	5.680478889	0.033000116	-1.093397778
TAS2R31	7.078646667	5.862375556	0.008510098	-1.216271111
TCF21	8.091003333	6.412915556	0.001125824	-1.678087778
TENM3	8.205303333	6.956973333	0.019408642	-1.24833
TFPI	8.6311	7.58293	0.005551188	-1.04817
THBD	8.354823333	7.284455556	0.004521404	-1.070367778
TMEM109	10.10218667	9.079675556	0.005472318	-1.022511111
TMEM26	7.271173333	6.16002	0.028884439	-1.111153333
TNXB	9.174756667	7.370361111	1.91E-05	-1.804395556
TSPAN7	10.44686	9.156041111	4.69E-05	-1.290818889
TTLL7	8.6943	7.166854444	0.000962706	-1.527445556
WEE1	9.66709	7.805838889	0.000623577	-1.861251111
WT1	10.10830333	9.030531111	0.039505784	-1.077772222
ZEB2	10.57323667	9.461408889	0.000304216	-1.111827778
ZFHX4	8.06592	6.567295556	0.004396215	-1.498624444
ZFPM2	9.931136667	8.174546667	0.00035081	-1.75659
ZNF204P	6.952393333	5.499644444	0.00293785	-1.452748889
ZNF676	8.7006	7.044083333	0.003749358	-1.656516667
ZNF860	6.751863333	5.646107778	0.000601986	-1.105755556
