# Supplementary material for: Different DNA methylome, transcriptome and histological features in uterine fibroids with and without MED12 mutations
Source: Sci Rep. 2022 May 26;12:8912. doi: 10.1038/s41598-022-12899-7 (PMC9135739; doi:10.1038/s41598-022-12899-7)
Supplement: Supplementary file 7 — Supplementary Table S5. [file 41598_2022_12899_MOESM7_ESM.pdf]

Different DNA methylome, transcriptome and histological features in uterine fibroids with and without MED12 mutations

Ryo Maekawa*, Department of Obstetrics and Gynecology, Yamaguchi University Graduate School of Medicine, Ube, 755-8505 Japan

Shun Sato, Department of Obstetrics and Gynecology, Yamaguchi University Graduate School of Medicine, Ube, 755-8505 Japan

Tetsuro Tamehisa, Department of Obstetrics and Gynecology, Yamaguchi University Graduate School of Medicine, Ube, 755-8505 Japan

Takahiro Sakai, Department of Obstetrics and Gynecology, Yamaguchi University Graduate School of Medicine, Ube, 755-8505 Japan

Takuya Kajimura, Department of Obstetrics and Gynecology, Yamaguchi University Graduate School of Medicine, Ube, 755-8505 Japan

Kotaro Sueoka, Department of Obstetrics and Gynecology, Yamaguchi University Graduate School of Medicine, Ube, 755-8505 Japan

Norihiro Sugino, Department of Obstetrics and Gynecology, Yamaguchi University Graduate School of Medicine, Ube, 755-8505 Japan

Supplemental Table S5. GO analysis with the genes whose promoters are differentially methylated between the MED12m-positive and -negative uterine fibroids.

DNA methylation status	Gene count	Gene name	GO enrichment analysis (p<0.05)
hypermethylated in MED12m-positive	25	DEF6 PF4 PLCE1 FLI1 SLC8A1 NAALAD2 FOXF2 PDGFRA SLC15A2 PRKCZ KCNA4 TLX1NB PCDHB7 PAPPA2 LOC654433 TUBGCP3 GIMAP2 PARP12 CNGA3 AMPD2 ITGBL1 OCIAD2 GRHL3 ZNF283 NUAK1	No significant GO terms
hypomethylated in MED12m-positive	73	ARHGEF4 LRRC2 MIR23B C9orf3 MIR27B MAFB FAM123C LEPREL1 CRYM GABRR1 NCF2 NFKBIL1 MYH11 NPAS4 NT5DC3 MIR24-1 C1QL1 GNG7 HOPX SPSB1 MGAT5B TRAF3IP2 EP400 RSPH6A FYN ANKRD11 SERPINA6 OTX1 KIAA0182 PLA1A TRIM26 CPLX2 BTBD2 ELFN1 ITFG2 SH3BP4 COL6A3 NCEH1 NNMT BEST1 SYCN LOC387646 MIR592 ZNF536 C6orf115 OSBPL8 ATP8B1 SPESP1 NOX5 STAT3 FCHO2 IGFN1 EFNB2 MIR874 HRNBP3 SOCS2 MEF2D EXPH5 LGALS7 CDH6 ELF1 MICAL2 ANKFY1	GO:0030154~cell differentiation
