# Supplementary material for: Different DNA methylome, transcriptome and histological features in uterine fibroids with and without MED12 mutations
Source: Sci Rep. 2022 May 26;12:8912. doi: 10.1038/s41598-022-12899-7 (PMC9135739; doi:10.1038/s41598-022-12899-7)
Supplement: Supplementary file 8 — Supplementary Table S6. [file 41598_2022_12899_MOESM8_ESM.pdf]

Different DNA methylome, transcriptome and histological features in uterine fibroids with and without MED12 mutations

Ryo Maekawa*, Department of Obstetrics and Gynecology, Yamaguchi University Graduate School of Medicine, Ube, 755-8505 Japan

Shun Sato, Department of Obstetrics and Gynecology, Yamaguchi University Graduate School of Medicine, Ube, 755-8505 Japan

Tetsuro Tamehisa, Department of Obstetrics and Gynecology, Yamaguchi University Graduate School of Medicine, Ube, 755-8505 Japan

Takahiro Sakai, Department of Obstetrics and Gynecology, Yamaguchi University Graduate School of Medicine, Ube, 755-8505 Japan

Takuya Kajimura, Department of Obstetrics and Gynecology, Yamaguchi University Graduate School of Medicine, Ube, 755-8505 Japan

Kotaro Sueoka, Department of Obstetrics and Gynecology, Yamaguchi University Graduate School of Medicine, Ube, 755-8505 Japan

Norihiro Sugino, Department of Obstetrics and Gynecology, Yamaguchi University Graduate School of Medicine, Ube, 755-8505 Japan

Supplemental Table S6. GO analysis with the genes whose promoters are differentially methylated among the subtypes of MED12m-negarive uterine fibroids.

DNA methylation status	Gene count	Gene name	GO enrichment analysis (p<0.05)
hypermethylated in subtype-1 compared to subtype-2	4	PLCE1 PAPPA2 GIMAP2 AMPD2	No significant GO terms
hypermethylated in subtype-1 compared to subtype-3	3	PLCE1 PAPPA2 GIMAP2	No significant GO terms
hypermethylated in subtype-2 compared to subtype-1	30	LRRC2 MIR23B C9orf3 MIR27B CRYM GABRR1 NFKBIL1 MYH11 NPAS4 NT5DC3 MIR24-1 TRAF3IP2 ELFN1 COL6A3 NCEH1 BEST1 SYCN LOC387646 C6orf115 ATP8B1 STAT3 FCHO2 IGFN1 BTBD2 ELF1 OTX1 ANKFY1 MARCH3 PCGF3 LUM	No significant GO terms
hypermethylated in subtype-2 compared to subtype-3	11	MIR23B C9orf3 MIR27B NT5DC3 KCNA4 BEST1 C6orf115 HRNBP3 CNGA3 PCGF3 LUM	No significant GO terms
hypermethylated in subtype-3 compared to subtype-1	20	LRRC2 LEPREL1 MIR24-1 C9orf3 SPSB1 EP400 ELFN1 COL6A3 NCEH1 NNMT ATP8B1 STAT3 FCHO2 IGFN1 EFNB2 SOCS2 BTBD2 MEF2D ELF1 ANKFY1	No significant GO terms
hypermethylated in subtype-3 compared to subtype-2	3	NCF2 PLA1A PCDHB7	No significant GO terms
