# Supplementary material for: Different DNA methylome, transcriptome and histological features in uterine fibroids with and without MED12 mutations
Source: Sci Rep. 2022 May 26;12:8912. doi: 10.1038/s41598-022-12899-7 (PMC9135739; doi:10.1038/s41598-022-12899-7)
Supplement: Supplementary file 9 — Supplementary Table S7. [file 41598_2022_12899_MOESM9_ESM.pdf]

Different DNA methylome, transcriptome and histological features in uterine fibroids with and without MED12 mutations

Ryo Maekawa*, Department of Obstetrics and Gynecology, Yamaguchi University Graduate School of Medicine, Ube, 755-8505 Japan

Shun Sato, Department of Obstetrics and Gynecology, Yamaguchi University Graduate School of Medicine, Ube, 755-8505 Japan

Tetsuro Tamehisa, Department of Obstetrics and Gynecology, Yamaguchi University Graduate School of Medicine, Ube, 755-8505 Japan

Takahiro Sakai, Department of Obstetrics and Gynecology, Yamaguchi University Graduate School of Medicine, Ube, 755-8505 Japan

Takuya Kajimura, Department of Obstetrics and Gynecology, Yamaguchi University Graduate School of Medicine, Ube, 755-8505 Japan

Kotaro Sueoka, Department of Obstetrics and Gynecology, Yamaguchi University Graduate School of Medicine, Ube, 755-8505 Japan

Norihiro Sugino, Department of Obstetrics and Gynecology, Yamaguchi University Graduate School of Medicine, Ube, 755-8505 Japan

Supplemental Table S7. GO enrichment analysis with the differently expressed genes between the subtype-1 of the MED12m-negative uterine fibroids and the myometrium.

GO term	P value (p<0.05)
Increased 171 genes	
GO:0006396~RNA processing	2.55E-101
GO:0002181~cytoplasmic translation	4.05E-04
GO:0006614~SRP-dependent cotranslational protein targeting to membrane	4.49E-04
GO:0019083~viral transcription	0.001162729
GO:0000184~nuclear-transcribed mRNA catabolic process, nonsense-mediated decay	0.001643678
GO:0006413~translational initiation	0.002413068
GO:0006364~rRNA processing	0.011902459
GO:0006412~translation	0.020622951
Decreased 188 genes	
GO:0007155~cell adhesion	5.09E-05
GO:0006954~inflammatory response	5.14E-04
GO:1902895~positive regulation of pri-miRNA transcription from RNA polymerase II promoter	5.48E-04
GO:0045429~positive regulation of nitric oxide biosynthetic process	6.45E-04
GO:0034097~response to cytokine	8.75E-04
GO:0007568~aging	9.41E-04
GO:0016055~Wnt signaling pathway	0.001557416
GO:0032496~response to lipopolysaccharide	0.001796186
GO:0001525~angiogenesis	0.004384783
GO:0098609~cell-cell adhesion	0.005021839
GO:0071277~cellular response to calcium ion	0.005998148
GO:0007010~cytoskeleton organization	0.006585854
GO:0007165~signal transduction	0.020518169
GO:0007596~blood coagulation	0.021752169
GO:0008285~negative regulation of cell proliferation	0.035365282
GO:0070374~positive regulation of ERK1 and ERK2 cascade	0.039652766
