# Supplementary material for: Different DNA methylome, transcriptome and histological features in uterine fibroids with and without MED12 mutations
Source: Sci Rep. 2022 May 26;12:8912. doi: 10.1038/s41598-022-12899-7 (PMC9135739; doi:10.1038/s41598-022-12899-7)
Supplement: Supplementary file 10 — Supplementary Table S8. [file 41598_2022_12899_MOESM10_ESM.pdf]

Different DNA methylome, transcriptome and histological features in uterine fibroids with and without MED12 mutations

Ryo Maekawa*, Department of Obstetrics and Gynecology, Yamaguchi University Graduate School of Medicine, Ube, 755-8505 Japan

Shun Sato, Department of Obstetrics and Gynecology, Yamaguchi University Graduate School of Medicine, Ube, 755-8505 Japan

Tetsuro Tamehisa, Department of Obstetrics and Gynecology, Yamaguchi University Graduate School of Medicine, Ube, 755-8505 Japan

Takahiro Sakai, Department of Obstetrics and Gynecology, Yamaguchi University Graduate School of Medicine, Ube, 755-8505 Japan

Takuya Kajimura, Department of Obstetrics and Gynecology, Yamaguchi University Graduate School of Medicine, Ube, 755-8505 Japan

Kotaro Sueoka, Department of Obstetrics and Gynecology, Yamaguchi University Graduate School of Medicine, Ube, 755-8505 Japan

Norihiro Sugino, Department of Obstetrics and Gynecology, Yamaguchi University Graduate School of Medicine, Ube, 755-8505 Japan

Supplemental Table S8. GO enrichment analysis with the differently expressed genes between the subtype-2 of the MED12m-negative uterine fibroids and the myometrium.

GO term	P value (p<0.05)
Increased 283 genes	
GO:0006396~RNA processing	3.35E-41
GO:0000184~nuclear-transcribed mRNA catabolic process, nonsense-mediated decay	2.65E-07
GO:0002181~cytoplasmic translation	1.31E-06
GO:0006614~SRP-dependent cotranslational protein targeting to membrane	1.59E-06
GO:0006413~translational initiation	4.97E-06
GO:0019083~viral transcription	9.11E-06
GO:0006412~translation	4.12E-04
GO:0006364~rRNA processing	6.20E-04
GO:0006355~regulation of transcription, DNA-templated	0.002003548
GO:0001822~kidney development	0.004096864
GO:0008584~male gonad development	0.011024651
GO:0007623~circadian rhythm	0.014159859
GO:0000398~mRNA splicing, via spliceosome	0.015455088
GO:0007050~cell cycle arrest	0.024022485
GO:0008380~RNA splicing	0.030497776
Decreased 366 genes	
GO:0030198~extracellular matrix organization	2.53E-05
GO:0045766~positive regulation of angiogenesis	6.82E-05
GO:0001523~retinoid metabolic process	7.95E-05
GO:0070373~negative regulation of ERK1 and ERK2 cascade	7.96E-05
GO:0045600~positive regulation of fat cell differentiation	1.92E-04
GO:0007165~signal transduction	3.13E-04
GO:0042059~negative regulation of epidermal growth factor receptor signaling pathway	3.15E-04
GO:1900026~positive regulation of substrate adhesion-dependent cell spreading	6.42E-04
GO:0006954~inflammatory response	9.95E-04
GO:0002576~platelet degranulation	0.001022669
GO:0098609~cell-cell adhesion	0.001186118
GO:0042572~retinol metabolic process	0.001539173
GO:0007155~cell adhesion	0.001816217
GO:0048662~negative regulation of smooth muscle cell proliferation	0.002423937
GO:0035556~intracellular signal transduction	0.002680805
GO:0010811~positive regulation of cell-substrate adhesion	0.003289171
GO:0030335~positive regulation of cell migration	0.003482585
GO:0060021~palate development	0.004401246
GO:0043627~response to estrogen	0.005321001
GO:0071364~cellular response to epidermal growth factor stimulus	0.007090592
GO:0001938~positive regulation of endothelial cell proliferation	0.007143175
GO:0042493~response to drug	0.007421376
GO:0007160~cell-matrix adhesion	0.007775914
GO:0006936~muscle contraction	0.008492016
GO:0032355~response to estradiol	0.010064133
GO:0006986~response to unfolded protein	0.012256185
GO:0051496~positive regulation of stress fiber assembly	0.012256185
GO:0030336~negative regulation of cell migration	0.014102931
GO:0042127~regulation of cell proliferation	0.016004853
GO:0022900~electron transport chain	0.01645628
GO:0051592~response to calcium ion	0.017391532
GO:0008285~negative regulation of cell proliferation	0.018154295
GO:0001525~angiogenesis	0.020169195
GO:0006629~lipid metabolic process	0.020838166
GO:0010595~positive regulation of endothelial cell migration	0.022561155
GO:0046718~viral entry into host cell	0.022906344
GO:0016477~cell migration	0.025854552
GO:0006805~xenobiotic metabolic process	0.027609236
GO:0030036~actin cytoskeleton organization	0.028160418
GO:0007166~cell surface receptor signaling pathway	0.029613318
GO:0008217~regulation of blood pressure	0.03122256
GO:0007229~integrin-mediated signaling pathway	0.032884727
GO:0008360~regulation of cell shape	0.038359495
GO:0051897~positive regulation of protein kinase B signaling	0.03974895
