# Supplementary material for: Different DNA methylome, transcriptome and histological features in uterine fibroids with and without MED12 mutations
Source: Sci Rep. 2022 May 26;12:8912. doi: 10.1038/s41598-022-12899-7 (PMC9135739; doi:10.1038/s41598-022-12899-7)
Supplement: Supplementary file 11 — Supplementary Table S9. [file 41598_2022_12899_MOESM11_ESM.pdf]

Different DNA methylome, transcriptome and histological features in uterine fibroids with and without MED12 mutations

Ryo Maekawa*, Department of Obstetrics and Gynecology, Yamaguchi University Graduate School of Medicine, Ube, 755-8505 Japan

Shun Sato, Department of Obstetrics and Gynecology, Yamaguchi University Graduate School of Medicine, Ube, 755-8505 Japan

Tetsuro Tamehisa, Department of Obstetrics and Gynecology, Yamaguchi University Graduate School of Medicine, Ube, 755-8505 Japan

Takahiro Sakai, Department of Obstetrics and Gynecology, Yamaguchi University Graduate School of Medicine, Ube, 755-8505 Japan

Takuya Kajimura, Department of Obstetrics and Gynecology, Yamaguchi University Graduate School of Medicine, Ube, 755-8505 Japan

Kotaro Sueoka, Department of Obstetrics and Gynecology, Yamaguchi University Graduate School of Medicine, Ube, 755-8505 Japan

Norihiro Sugino, Department of Obstetrics and Gynecology, Yamaguchi University Graduate School of Medicine, Ube, 755-8505 Japan

Supplemental Table S9. GO enrichment analysis with the differently expressed genes between the subtype-3 of the MED12m-negative uterine fibroids and the myometrium.

GO term	P value (p<0.05)
Increased 96 genes	
GO:0006396~RNA processing	2.20E-27
GO:0006412~translation	0.012232755
Decreased 129 genes	
GO:0042572~retinol metabolic process	1.09E-05
GO:0001523~retinoid metabolic process	3.03E-05
GO:0070373~negative regulation of ERK1 and ERK2 cascade	0.001419186
GO:0006954~inflammatory response	0.002014036
GO:0006869~lipid transport	0.002292833
GO:0045944~positive regulation of transcription from RNA polymerase II promoter	0.002916542
GO:0098609~cell-cell adhesion	0.004540914
GO:0055085~transmembrane transport	0.00798687
GO:0032496~response to lipopolysaccharide	0.011509115
GO:0030336~negative regulation of cell migration	0.011761895
GO:0007155~cell adhesion	0.012698665
GO:0007165~signal transduction	0.03841679
GO:0007275~multicellular organism development	0.039038578
