# Supplementary material for: Different DNA methylome, transcriptome and histological features in uterine fibroids with and without MED12 mutations
Source: Sci Rep. 2022 May 26;12:8912. doi: 10.1038/s41598-022-12899-7 (PMC9135739; doi:10.1038/s41598-022-12899-7)
Supplement: Supplementary file 12 — Supplementary Table S10. [file 41598_2022_12899_MOESM12_ESM.pdf]

Different DNA methylome, transcriptome and histological features in uterine fibroids with and without MED12 mutations

Ryo Maekawa*, Department of Obstetrics and Gynecology, Yamaguchi University Graduate School of Medicine, Ube, 755-8505 Japan

Shun Sato, Department of Obstetrics and Gynecology, Yamaguchi University Graduate School of Medicine, Ube, 755-8505 Japan

Tetsuro Tamehisa, Department of Obstetrics and Gynecology, Yamaguchi University Graduate School of Medicine, Ube, 755-8505 Japan

Takahiro Sakai, Department of Obstetrics and Gynecology, Yamaguchi University Graduate School of Medicine, Ube, 755-8505 Japan

Takuya Kajimura, Department of Obstetrics and Gynecology, Yamaguchi University Graduate School of Medicine, Ube, 755-8505 Japan

Kotaro Sueoka, Department of Obstetrics and Gynecology, Yamaguchi University Graduate School of Medicine, Ube, 755-8505 Japan

Norihiro Sugino, Department of Obstetrics and Gynecology, Yamaguchi University Graduate School of Medicine, Ube, 755-8505 Japan

Supplemental Table S10. Increased DNA methylation and mRNA expression levels of SATB2 or NRG1 in uterine fibroids with and without MED12 mutations.

	MED12m-positive uterine fibroids (n = 9)	MED12m-negative uterine fibroids (n = 12)	Total
DNA methylation (15% or more DNA methylation level than myometrium)			
SATB2	88.9% (8/9)	75% (9/12)	81% (17/21)
NRG1	100% (9/9)	75% (9/12)	85.7% (18/21)
mRNA expression (2-fold or more mRNA expression level than myometrium)			
SATB2	100% (9/9)	75% (9/12)	85.7 % (18/21)
NRG1	100% (9/9)	66.7% (8/12)	81.0 % (17/21)
