# Supplementary material for: Different DNA methylome, transcriptome and histological features in uterine fibroids with and without MED12 mutations
Source: Sci Rep. 2022 May 26;12:8912. doi: 10.1038/s41598-022-12899-7 (PMC9135739; doi:10.1038/s41598-022-12899-7)
Supplement: Supplementary file 13 — Supplementary Table 11. [file 41598_2022_12899_MOESM13_ESM.pdf]

Different DNA methylome, transcriptome and histological features in uterine fibroids with and without MED12 mutations

Ryo Maekawa*, Department of Obstetrics and Gynecology, Yamaguchi University Graduate School of Medicine, Ube, 755-8505 Japan

Shun Sato, Department of Obstetrics and Gynecology, Yamaguchi University Graduate School of Medicine, Ube, 755-8505 Japan

Tetsuro Tamehisa, Department of Obstetrics and Gynecology, Yamaguchi University Graduate School of Medicine, Ube, 755-8505 Japan

Takahiro Sakai, Department of Obstetrics and Gynecology, Yamaguchi University Graduate School of Medicine, Ube, 755-8505 Japan

Takuya Kajimura, Department of Obstetrics and Gynecology, Yamaguchi University Graduate School of Medicine, Ube, 755-8505 Japan

Kotaro Sueoka, Department of Obstetrics and Gynecology, Yamaguchi University Graduate School of Medicine, Ube, 755-8505 Japan

Norihiro Sugino, Department of Obstetrics and Gynecology, Yamaguchi University Graduate School of Medicine, Ube, 755-8505 Japan

Supplemental Table S11. PCR primers for combined bisulfite restriction analysis (COBRA) and quantitative real-time RT-PCR (qRT-PCR)

Gene name	Usage	Amplified region (UCSC hg19)	PCR primers	Product size (bp)	# of CpGs	Restriction enzyme<br>Fragment size (bp)
SATB2	COBRA	chr2: 200,330,754-200,331,048	F: TTAGAAAAGTGGTGGGTTTTTTTTAGTT<br>R: TACACCCACAACTCAAATTCTACTATCC	295	9	*Hpy*CH4IV<br>148 / 147
NRG1	COBRA	chr8: 32,503,979-32,504,264	F: TGTTGTTGAAAAGTTTATTTTTTTAGGGAG<br>R: ACATCAATTAAACCTAATCCCCATAACC	286	3	*Taq* I<br>132 / 154
SATB2	real-time RT-PCR	chr2: 200,193,607-200,213,649	F: GATAAACCAACAGATTGCCGTTAG<br>R: CTTACGCAGAATCTCAGACAACAAT	253	-	-
NRG1	real-time RT-PCR	chr8: 32,453,412-32,463,168	F: GTGTGAAACCAGTTCTGAATACTCC	200	-	-
